# Differential localization of LTA synthesis proteins and their interaction with the cell division machinery in *Staphylococcus aureus*

**DOI:** 10.1111/mmi.12551

**Published:** 2014-03-20

**Authors:** Nathalie T Reichmann, Carolina Piçarra Cassona, João M Monteiro, Amy L Bottomley, Rebecca M Corrigan, Simon J Foster, Mariana G Pinho, Angelika Gründling

**Affiliations:** 1 Section of Microbiology and MRC Centre for Molecular Bacteriology and Infection, Imperial College LondonLondon, SW7 2AZ, UK; 2 Instituto de Technologia Química e Biológica, Universidade Nova de LisboaOeiras, Portugal; 3 The Krebs Institute, Department of Molecular Biology and Biotechnology, University of SheffieldSheffield, UK

## Abstract

Lipoteichoic acid (LTA) is an important cell wall component of Gram-positive bacteria. In *Staphylococcus aureus* it consists of a polyglycerolphosphate-chain that is retained within the membrane via a glycolipid. Using an immunofluorescence approach, we show here that the LTA polymer is not surface exposed in *S. aureus*, as it can only be detected after digestion of the peptidoglycan layer. *S. aureus* mutants lacking LTA are enlarged and show aberrant positioning of septa, suggesting a link between LTA synthesis and the cell division process. Using a bacterial two-hybrid approach, we show that the three key LTA synthesis proteins, YpfP and LtaA, involved in glycolipid production, and LtaS, required for LTA backbone synthesis, interact with one another. All three proteins also interacted with numerous cell division and peptidoglycan synthesis proteins, suggesting the formation of a multi-enzyme complex and providing further evidence for the co-ordination of these processes. When assessed by fluorescence microscopy, YpfP and LtaA fluorescent protein fusions localized to the membrane while the LtaS enzyme accumulated at the cell division site. These data support a model whereby LTA backbone synthesis proceeds in *S. aureus* at the division site in co-ordination with cell division, while glycolipid synthesis takes place throughout the membrane.

## Introduction

A polyglycerolphosphate (PGP)-type of LTA is found in a variety of Gram-positive bacteria and its synthesis and function has been studied most extensively in *S. aureus* (Fischer, 1990; Rahman *et al*., 2009; Reichmann and Gründling, 2011). In this organism, the PGP backbone chain is decorated with d-alanine esters and linked to the outside of the membrane through a diglucosyldiacylglycerol (Glc_2_-DAG) glycolipid anchor (Duckworth *et al*., 1975; Fischer and Rosel, 1980; Kiriukhin *et al*., 2001; Gründling and Schneewind, 2007a). LTA production begins in the cytoplasm of the cell with the synthesis of Glc_2_-DAG (Kiriukhin *et al*., 2001; Gründling and Schneewind, 2007a). The processive glycosyltransferase YpfP transfers two glucose moieties from the nucleotide activated sugar UDP-glucose (UDP-Glc) to the membrane lipid diacylglycerol (DAG) to generate Glc_2_-DAG (Jorasch *et al*., 2000; Kiriukhin *et al*., 2001; Fedtke *et al*., 2007). Next, the multimembrane spanning protein LtaA is thought to translocate the glycolipid from the inner to the outer leaflet of the membrane (Gründling and Schneewind, 2007a). Lastly, the lipoteichoic acid synthase LtaS polymerizes the PGP backbone chain whereby the glycerolphosphate subunits are derived from the head group of the membrane lipid phosphatidylglycerol (Koch *et al*., 1984; Fischer, 1994; Gründling and Schneewind, 2007b; Karatsa-Dodgson *et al*., 2010). Together, the three key enzymes YpfP, LtaA and LtaS are therefore responsible for the synthesis of the core LTA structure in *S. aureus*.

During or after polymerization of the PGP backbone chain, the glycerolphosphate subunits are further decorated with d-alanine esters (Fischer and Rosel, 1980; Neuhaus and Baddiley, 2003). Four proteins, DltA-D, encoded in the *dlt* operon are essential for this process (Neuhaus *et al*., 1996). DltA, a d-alanine-d-alanyl carrier protein ligase, is responsible for the ligation of d-alanine to the d-alanyl carrier protein DltC (Heaton and Neuhaus, 1992; 1994[Bibr b16],[Bibr b17]). How the d-alanine residues are subsequently linked to the PGP backbone chain and the roles of the remaining two proteins, DltB and DltD, in this process have not been fully elucidated. However, based on recent findings, it is likely that the d-alanylation of LTA proceeds as originally proposed by Fischer and colleagues through a lipid-linked undecaprenyl phosphate (C_55_-P) intermediate (Perego *et al*., 1995; Reichmann *et al*., 2013). In such a model, DltB facilitates the transfer of d-alanines from DltC to C_55_-P to produce D-Ala-P-C_55_ and possibly the subsequent transfer of this lipid-linked intermediate across the membrane. Following this, DltD, which has an N-terminus in and C-terminus out membrane topology, functions on the outside of the cell and aids in the final transfer step of d-alanine onto LTA (Perego *et al*., 1995; Reichmann *et al*., 2013).

In the rod-shaped bacteria *Bacillus subtilis* and *Listeria monocytogenes*, the absence of LTA results in a severe filamentation phenotype, highlighting a requirement of LTA for proper cell division in these bacteria (Schirner *et al*., 2009; Webb *et al*., 2009; Wörmann *et al*., 2011a). This is further emphasized by the observation that the *B. subtilis* glycosyltransferase UgtP, the homologue of the *S. aureus* YpfP protein, as well as LtaS and YqgS, two of the four LtaS homologues, localize to the cell division site in this organism (Nishibori *et al*., 2005; Weart *et al*., 2007; Schirner *et al*., 2009). Furthermore, it has been shown that the cell division protein FtsZ delocalizes in a filamenting *B. subtilis ltaS* mutant strain (Schirner *et al*., 2009). In studies aimed at identifying determinants involved in nutrient-dependent cell size control, an additional link between glycolipid synthesis enzyme UgtP, cell size and cell division was found (Weart *et al*., 2007; Chien *et al*., 2012). It was reported that *B. subtilis* strains with mutations in *ugtP* (*ypfP*) or *pgcA*, encoding an enzyme required for the production of glucose 1-phosphate, a precursor of the YpfP substrate UDP-Glc, are smaller in size as compared with a wild type control strain (Weart *et al*., 2007). UgtP forms oligomers and interacts with FtsZ, inhibiting its polymerization in an *in vitro* assay system (Weart *et al*., 2007; Chien *et al*., 2012). Binding of UDP-Glc to UgtP shifts the equilibrium from an UgtP self-interaction towards an interaction with FtsZ, thus reducing the availability of FtsZ for the assembly of the cytokinetic ring and resulting in a delay in cell division and increase in cell size (Chien *et al*., 2012). A second group also reported that a *B. subtilis ugtP* mutant strain has shorter cells in stationary phase (Matsuoka *et al*., 2011). During the exponential growth phase the mutant cells were found to be fatter and to display a curved and filamentous morphology (Matsuoka *et al*., 2011). Regardless of the slight differences in phenotypic observations, taken together these studies provide experimental evidence for a physical interaction between the LTA glycolipid anchor synthesis enzyme UgtP and the cell division machinery in *B. subtilis.*

Currently, no information is available on the localization of LTA synthesis proteins in *S. aureus* and no studies have been performed to investigate an interaction between LTA synthesis and cell division proteins in this organism. However, consistent with an interplay between these two processes in this organism, it has been documented that the absence of LTA leads to growth arrest in *S. aureus*, aberrant positioning of septa, enlargement of cells and eventual cell lysis (Gründling and Schneewind, 2007b; Oku *et al*., 2009). Furthermore, *S. aureus* mutants with defects in LTA glycolipid anchor production display numerous phenotypic alterations including cell morphology defects and an increased autolysis rate (Kiriukhin *et al*., 2001; Fedtke *et al*., 2007).

Using two different *S. aureus* strains, we show here that LTA is not a surface exposed molecule as often depicted. To further investigate the connection between cell division and LTA synthesis, protein–protein interaction and localization studies were performed. It was found that the three key LTA synthesis enzymes, YpfP, LtaA and LtaS, interact with one another, as well as with numerous cell division and peptidoglycan synthesis proteins, indicating the formation of a multi-enzyme complex. Using fluorescent protein fusions and microscopy analysis, it was shown that YpfP and LtaA localize to the membrane of *S. aureus*, while LtaS accumulates at the cell division site. This suggests that while glycolipid synthesis takes place throughout the cell membrane, LTA backbone synthesis occurs mainly at the division site. Taken together, this study provides new insight into the architecture of the Gram-positive cell wall and provides further experimental evidence for a coordination between the processes of cell division and LTA synthesis.

## Results

### Interactions between LTA synthesis, cell division and peptidoglycan synthesis proteins suggest the formation of a multiprotein complex

**Fig. 1:**
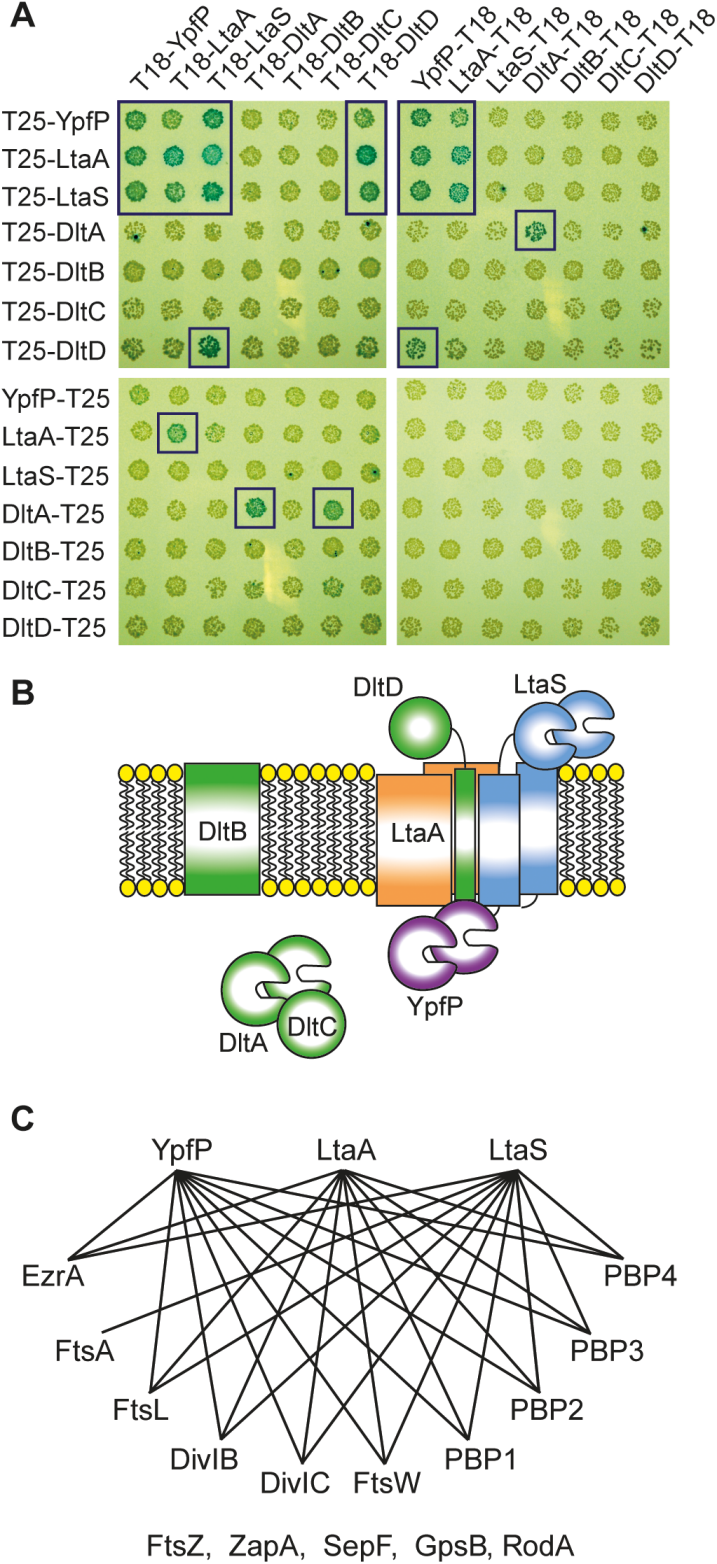
Protein–protein interactions between *S. aureus* LTA synthesis, cell division and peptidoglycan synthesis proteins.A. Protein–protein interactions between the *S. aureus* LTA synthesis proteins were tested using the BACTH system. *E. coli* BTH101 cells were co-transformed with plasmids expressing the indicated T25 and T18 fusion proteins and spotted onto IPTG and X-gal containing plates. The plates were incubated for 36–44 h at 30°C and images taken. Transformation reactions yielding blue colonies (further highlighted by a blue box) indicate a positive interaction.B. Schematic representation summarizing the protein–protein interactions detected in the BACTH assay shown in panel A.C. Schematic representation of the interactions between the core *S. aureus* LTA synthesis proteins and cell division and peptidoglycan synthesis proteins. Positive interactions are depicted as black lines in this representation. Proteins also tested using the two-hybrid assay, which did not show any interaction with the core LTA synthesis proteins are listed in the bottom row. Images of the actual transformation plates are shown in Fig. S1.

To investigate whether the *S. aureus* proteins involved in LTA synthesis might form a multi-enzyme complex, interactions between the core LTA synthesis proteins YpfP, LtaA and LtaS and the four proteins, DltA-D, required for the incorporation of d-alanines, were investigated using the bacterial adenylate cyclase two-hybrid (BACTH) system. To this end, plasmids for the expression of N- or C-terminal fusions between the LTA synthesis proteins and the T25 and T18 fragments of the adenylate cyclase were constructed and co-transformed into the adenylate cyclase negative *E. coli* strain BTH101. Several transformation reactions yielded blue colonies on X-gal and IPTG containing plates indicating an interaction between the two co-expressed proteins (Fig. 1A). Specifically, the results of this two-hybrid analysis highlighted self-interactions for YpfP, LtaA and LtaS as well as interactions with one another, indicating that the three core LTA synthesis proteins potentially form a multiprotein complex (Fig. 1A and B). During the d-alanylation process of LTA, d-alanine is transferred from the d-alanine-d-alanyl carrier protein ligase DltA to the carrier protein DltC (Heaton and Neuhaus, 1992; 1994). As expected, an interaction between these proteins was detected using the two-hybrid assay system (Fig. 1A and B). Furthermore, a DltA self-interaction was detected and DltD was found to interact with the core LTA synthesis proteins (Fig. 1A and B).

LTA depletion in *S. aureus* results in aberrant positioning of the septa, cell enlargement and eventual cell lysis, indicating a role for this polymer in both cell growth and cell division (Gründling and Schneewind, 2007b; Oku *et al*., 2009). In order to investigate this link further, the BACTH system was also employed to determine whether proteins involved in these different pathways could potentially physically interact. Proteins included in this analysis were the early stage cell division proteins FtsZ, EzrA, FtsA and ZapA, the late stage cell division proteins SepF, FtsL, GpsB, DivIB and DivIC, as well as peptidoglycan synthesis proteins RodA, FtsW, PBP1, PBP2, PBP3 and PBP4. As shown in Fig. S1 and summarized in Fig. 1C, the three core LTA synthesis proteins YpfP, LtaA and LtaS showed multiple interactions with early and late stage cell division proteins as well as the peptidoglycan synthesis proteins. These data provide initial evidence that the *S. aureus* LTA synthesis proteins form a multi-enzyme complex and physically interact with the cell division and peptidoglycan synthesis machineries and present the foundation for future protein–protein interaction studies directly in *S. aureus.*

### Generation of fluorescent protein fusions to the core *S. aureus* LTA synthesis proteins

**Fig. 2:**
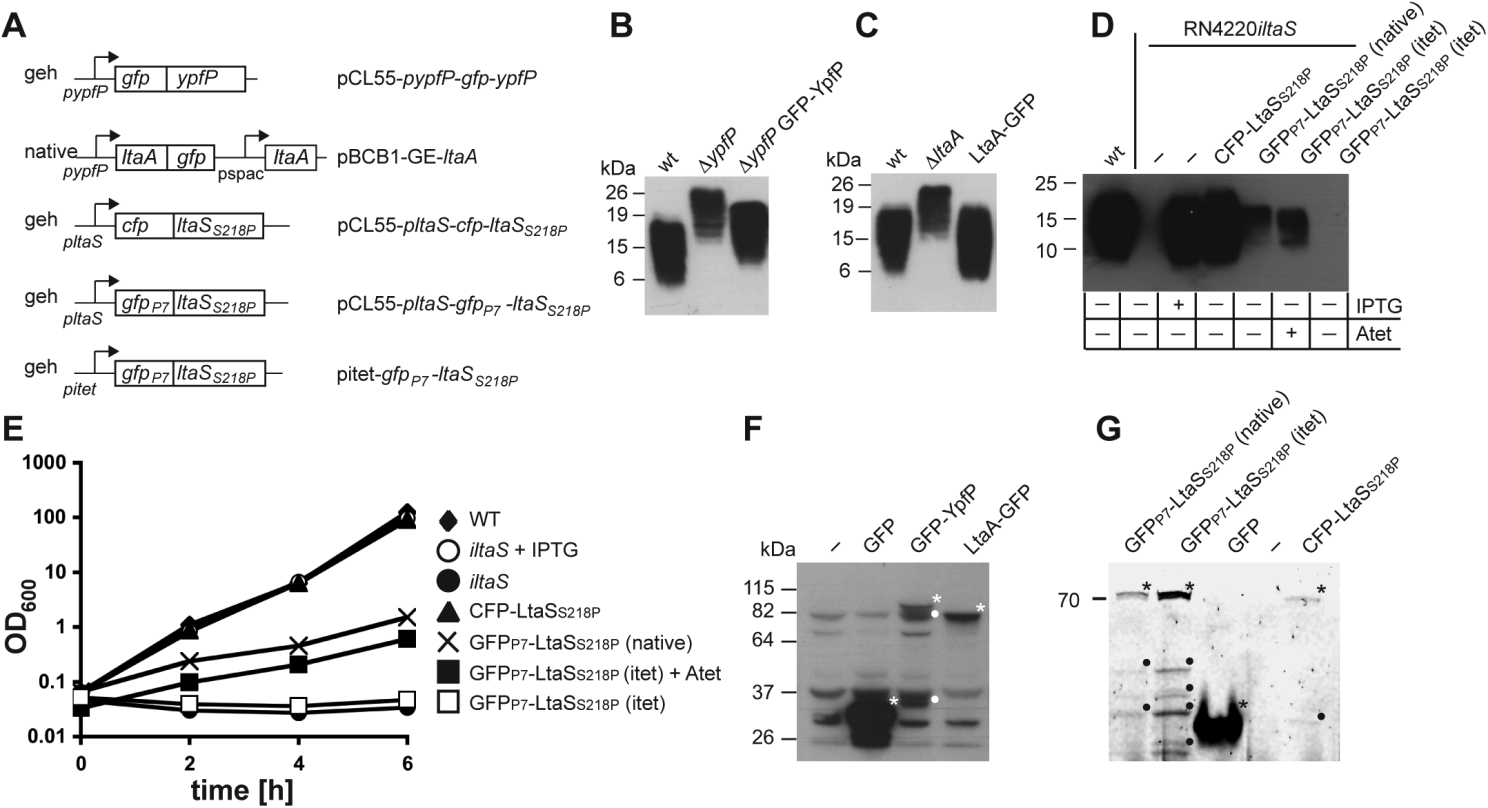
Complementation analysis using LTA synthesis-fluorescent protein fusions.A. Schematic representation of constructs for the expression of the fluorescent protein fusions. GFP–YpfP and LtaA–GFP fusion proteins were expressed from their native *ypfP* promoter from the *geh* locus or the native chromosomal location, respectively. The CFP–LtaS_S218P_ fusion was expressed from the native *ltaS* promoter and the GFP_P7_–LtaS_S218P_ fusion was expressed either from the native or the Atet inducible *itet* promoter from the *geh* locus.B–D. LTA analysis by Western blot. LTA was extracted from *S. aureus* strains and analysed by Western blot (B) RN4220Δ*spa* p*itet* (WT) RN4220Δ*spa*Δ*ypfP* p*itet* (Δ*ypfP*) or RN4220Δ*spa*Δ*ypfP* pCL55–p*ypfP*–*gfp–ypfP* (Δ*ypfP* GFP–YpfP), (C) the *S. aureus* strains RN4220Δ*spa* (WT), RN4220Δ*spa*Δ*ltaA* (Δ*ltaA*) and RN4220Δ*spa* pBCB1-GE-*ltaA* (LtaA–GFP), (D) and the *S. aureus* strains RN4220 pCL55 (WT), RN4220*iltaS* pCL55 (−), RN4220*iltaS* pCL55–p*ltaS–cfp–ltaS*_S218P_ (CFP–LtaS_S218P_), RN4220*iltaS* pCL55–p*ltaS–gfp_P7_–ltaS*_S218P_ [GFP_P7_–LtaS_S218P_ (native)] and RN4220*iltaS* p*itet–gfp_P7_–ltaS*_S218P_ [GFP_P7_–LtaS_S218P_ (itet)] in the presence and absence of 1 mM IPTG and 200 ng ml^−1^ Atet.E. Bacterial growth curves. Overnight cultures of *S. aureus* strains RN4220 pCL55 (WT; black diamonds), RN4220*iltaS* (*iltaS*; with 1 mM IPTG white circles or without IPTG black circles) or RN4220*iltaS* with pCL55–p*ltaS–cfp–ltaS*_S218P_ (CFP–LtaS_S218P_; black triangle), pCL55–p*ltaS*–*gfp_P7_*–*ltaS_S218P_* [GFP_P7_–LtaS_S218P_ (native); cross], p*itet*–*gfp_P7_*–*ltaS_S218P_* [GFP_P7_–LtaS_S218P_ (itet); with Atet black squares or without Atet white squares] were back-diluted to an OD_600_ of 0.07 into TSB medium where indicated with the appropriate inducer and grown for 4h. At this point (*T* = 0) all strains were back-diluted again 100-fold and the OD_600_ was subsequently measured every two hours for six hours.F–G. Detection of fluorescent protein fusions by Western blot or fluorescence imaging. (F) LAC* strains containing the integration vector pCL55 (−), pCL55–p*tet*–*gfpmut2* (GFP), pCL55–p*ypfP*–*gfp*–*ypfP* (GFP–YpfP) or pBCB1-GE-*ltaA* (LtaA–GFP) were grown to mid-exponential phase and samples prepared and analyzed by Western blot using an anti-GFP antibody. (G) LAC* strains containing the integration vector pCL55–p*ltaS*–*gfp_P7_*–*ltaS_S218P_* [GFP_P7_–LtaS_S218P_ (native)], p*itet*–*gfp_P7_*–*ltaS_S218P_* [GFP_P7_–LtaS_S218P_ (itet) grown in the presence of 200 ng ml^−1^ Atet], pCL55–p*tet*–*gfp* (GFP), no vector (−) or pCL55–p*ltaS*–*cfp*–*ltaS_S218P_* (CFP–LtaS_S218P_) were grown to mid-exponential phase and analysed by fluorescence imaging as described in the *Experimental procedures* section. Full-length fusion proteins are indicated with asterisks and degradation products with dots.

The *B. subtilis* homologues of YpfP and LtaS have been shown to localize to the septum in this organism (Weart *et al*., 2007; Schirner *et al*., 2009). Based on this observation and the interaction studies described above, it was decided to investigate the localization of the LTA synthesis proteins in *S. aureus*. To this end, YfpP, LtaA and LtaS fusions with different fluorescent proteins were generated (Fig. 2A). Initially the fusions were expressed in the *S. aureus* laboratory strain RN4220, for which appropriate mutant strains are available to test their functionality. Specifically, the green fluorescent protein (GFP) was fused to the N-terminal end of YpfP and the C-terminal end of LtaA, separated by a 3× EAAAK flexible amino acid linker region. The *ypfP* and *ltaA* genes are encoded within an operon, therefore both fusions were expressed from the native *ypfP* promoter using established chromosomal integration vectors that insert either into the ectopic lipase gene locus *geh* (GFP–YpfP) or the native locus (LtaA–GFP) (Fig. 2A). Due to defects in glycolipid synthesis or transport, the LTA polymer in *S. aureus ypfP* and *ltaA* mutants is linked to the membrane predominantly by diacylglycerol (DAG) and shows a slower mobility on SDS-PAGE gels, which can be readily visualized by Western blot analysis (Gründling and Schneewind, 2007a). Therefore, using appropriate *S. aureus* strains and this phenotypic alteration, the functionality of the GFP fusion proteins can be assessed in complementation assays. Expression of the GFP–YpfP or LtaA–GFP reverted the LTA production defect to near or complete wild type levels, suggesting that both fusion proteins are functional (Fig. 2B and C).

LtaS consists of five transmembrane helices linked to an extracellular enzymatic domain, which is cleaved by the signal peptidase SpsB and released into the culture supernatant (Wörmann *et al*., 2011b). Due to this processing step, fluorescent protein fusions to wild type LtaS were found to be very unstable and resulted in the accumulation of cleaved products in the cytoplasm of the cell, which prevented protein localization studies. Therefore, fluorescent protein fusions were produced using a previously described functional but less cleaved LtaS_S218P_ variant (Wörmann *et al*., 2011b). More specifically, three different fusion constructs were produced: a CFP–LtaS_S218P_ fusion expressed from the native *ltaS* promoter as well as a fusion to the fast folding GFP variant P7 (GFP_P7_–LtaS_S218P_), which was either expressed from the native *ltaS* promoter or the anhydrotetracycline (Atet) inducible promoter P*itet* (Fig. 2A). All fusions were expressed in *S. aureus* from a chromosomal integration vector. Complementation analysis revealed that the CFP–LtaS_S218P_ fusion was fully functional as its expression complemented growth and LTA production of an *S. aureus* strain with inducible *ltaS* expression, which does not grow or produce LTA in the absence of the inducer IPTG (Fig. 2D and E). The GFP_P7_–LtaS_S218P_ fusion showed partial functionality as its expression from the native or the inducible promoter (in the presence but not absence of Atet) in the inducible *ltaS* strain restored growth and LTA production to some extent (Fig. 2D and E).

Once constructs for the expression of LTA synthesis-fluorescent protein fusions were made, they were moved from the laboratory strain RN4220 into the clinically relevant methicillin resistant *S. aureus* strain LAC* (Boles *et al*., 2010). The expression and stability of the fluorescent protein fusions in LAC* was assessed by Western blot analysis with an anti-GFP antibody or using a fluorescence imager. Full-length fusion proteins could be detected for all fusions and some protein degradation was noted for the GFP–YpfP fusion and the GFP_P7_ and CFP fusions to LtaS_S218P_ (Fig. 2F and G).

### YpfP and LtaA localize to the cell membrane in *S. aureus*

**Fig. 3:**
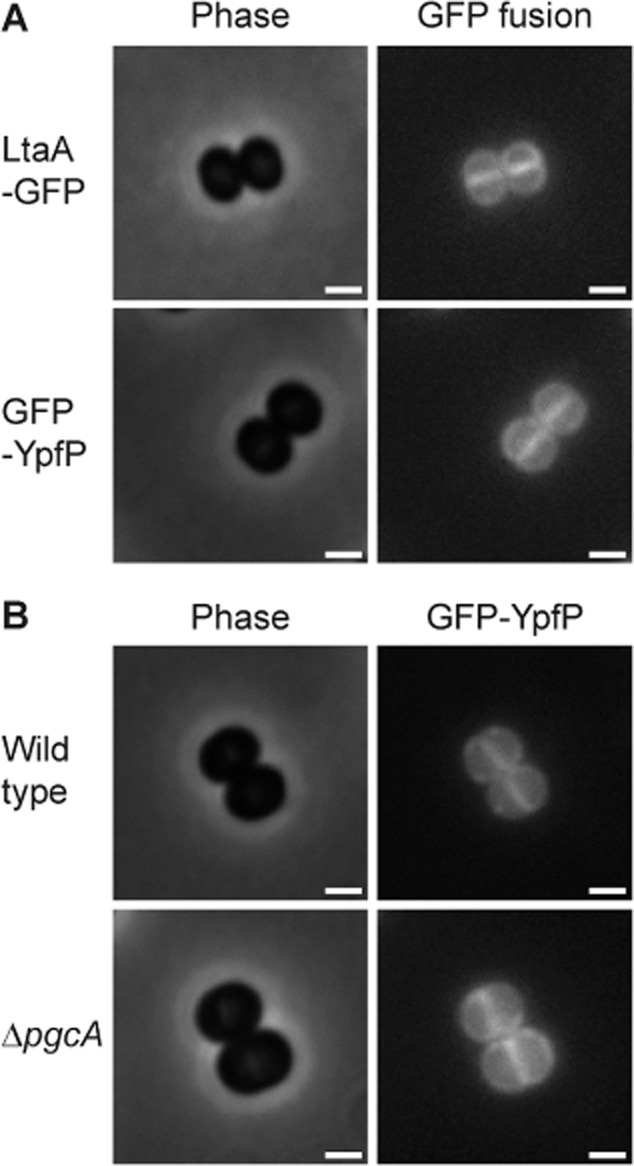
Localization of YpfP and LtaA in *S. aureus*. (A) *S. aureus* LAC* strains expressing the LtaA–GFP or GFP–YpfP fusion and (B) RN4220Δ*spa* and RN4220Δ*spa*Δ*pgcA* strains expressing the GFP–YpfP fusion protein were grown to mid-exponential phase, mounted on a 1.2% PBS agarose slide and subsequently observed by fluorescence microscopy. Phase contrast (left) and fluorescence (right) images are shown. Scale bar = 1 μm. Larger fields of cells are shown in Figs S2 and S4.

**Fig. 4:**
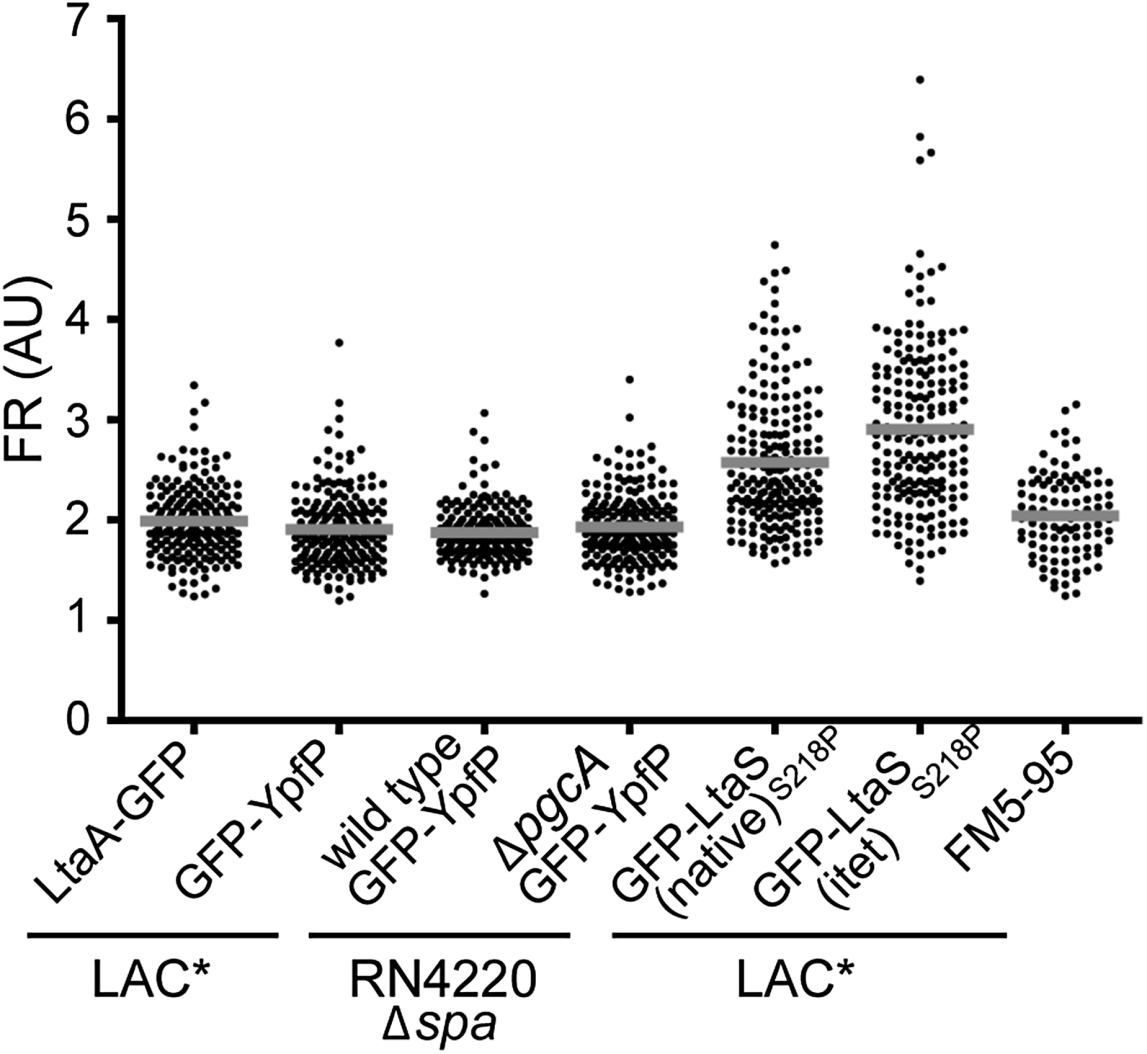
Determination of the fluorescence ratios (FRs) for the localization of LtaA, YpfP and LtaS. *S. aureus* strains were grown to mid-exponential phase, mounted on a 1.2% PBS agarose slide and observed by fluorescence microscopy. The fluorescence ratio (FR) between the signal obtained at the division site and the signal obtained at the lateral wall was determined for cells with complete septa as outlined by Atilano *et al*. (2010). FR values were plotted on a scatter graph and the average values are represented by the grey horizontal lines. The FRs of 200 cells with complete septa for strains LAC* pBCB1-GE-*ltaA* (LtaA–GFP; 1.99 ± 0.35), LAC* pCL55-p*ypfP*–*gfp*–*ypfP* (GFP–YpfP; 1.91 ± 0.37), RN4220Δ*spa* pCL55-p*ypfP*–*gfp*–*ypfP* (wild type GFP–YpfP (1.88 ± 0.25), RN4220Δ*spa*Δ*pgcA* pCL55-p*ypfP*–*gfp*–*ypfP* (Δ*pgcA* GFP-YpfP; 1.93 ± 0.34), LAC* pCL55–p*ltaS–gfp_p7_–ltaS*_S__218__P_ [GFP– *ltaS*_S__218__P_ (native); 2.57 ± 0.66] and LAC* p*itet–gfp_p7_–ltaS*_S__218__P_ [GFP–*ltaS*_S__218__P_ (itet); 2.91 ± 0.83] are plotted from left to right. The FRs of 100 cells of LAC* p*itet* stained with the membrane dye FM5–95 yielded an average FR value of 2.04 ± 0.42.

The localization of YpfP, a predicted cytoplasmic protein, and LtaA, a predicted multimembrane spanning protein required for glycolipid synthesis and transport were assessed by fluorescence microscopy. LtaA–GFP localized uniformly to the membrane, as expected (Figs 3A and S2). While somewhat spotty, YpfP also localized to the membrane, and did not appear to localize specifically to the division site (Figs 3A and S2). This is in contrast to *B. subtilis* where the YpfP homologue, UgtP, was found to accumulate specifically at the cell division site (Nishibori *et al*., 2005; Weart *et al*., 2007). To verify this localization pattern, cells with fully formed septa were analysed further to determine the fluorescence ratio (FR) of the signal at the septal and lateral membrane, as outlined in Atilano *et al*. (2010). Due to the formation of a double membrane at the division site, uniformly distributed fluorescently tagged membrane proteins will show twice the fluorescence signal at the septum compared with the lateral wall and a FR of approximately 2 will be obtained. In contrast, septal-localized proteins would exhibit a FR significantly higher than 2. As a control for this methodology, the FR of cells stained with the membrane dye FM5–95 was determined and found to be 2.04 ± 0.42 (Fig. 4, last column). FRs for LAC* expressing LtaA–GFP or GFP–YpfP were determined as 1.99 ± 0.35 and 1.91 ± 0.37 (Fig. 4), confirming that these proteins did not accumulate at the septum.

### The localization of YpfP is independent of its cytoplasmic substrate

As mentioned above, the *B. subtilis* YpfP homologue, UgtP, was shown to localize to the septum, though the absence of its cytoplasmic substrate UDP-Glc resulted in delocalization to randomly distributed spots (Weart *et al*., 2007). Removal of UDP-Glc can be achieved by mutating the *pgcA* gene, encoding for the α-phosphoglucomutase responsible for the conversion of glucose 6-phosphate to glucose 1-phosphate, a precursor used for the production of UDP-Glc (Lazarevic *et al*., 2005). In a similar manner, it was decided to investigate the effect of the absence of UDP-Glc on the localization of YpfP in *S. aureus* using an available *pgcA* mutant in an RN4220-derived *S. aureus* background strain. We confirmed the lack of phosphoglucomutase activity in the *pgcA* mutant strain (Fig. S3). Furthermore, in previous work, it was shown that strain RN4220Δ*spa*Δ*pgcA* is unable to produce glycolipids (Gründling and Schneewind, 2007a), all indicating the absence of UDP-Glc in this strain. As shown in Figs 3B and S4, both RN4220Δ*spa* (wild type) and the RN4220Δ*spa*Δ*pgcA* mutant displayed similar GFP–YpfP localization patterns, indicating that YpfP localization in *S. aureus* is independent of PgcA and hence the production of UDP-Glc. This was further confirmed by quantification of the fluorescence signal, where FRs of 1.88 ± 0.25 and 1.93 ± 0.34 were obtained for the wild type and the Δ*pgcA* mutant strain respectively (Fig. 4).

### LtaS accumulates at the cell division site

**Fig. 5:**
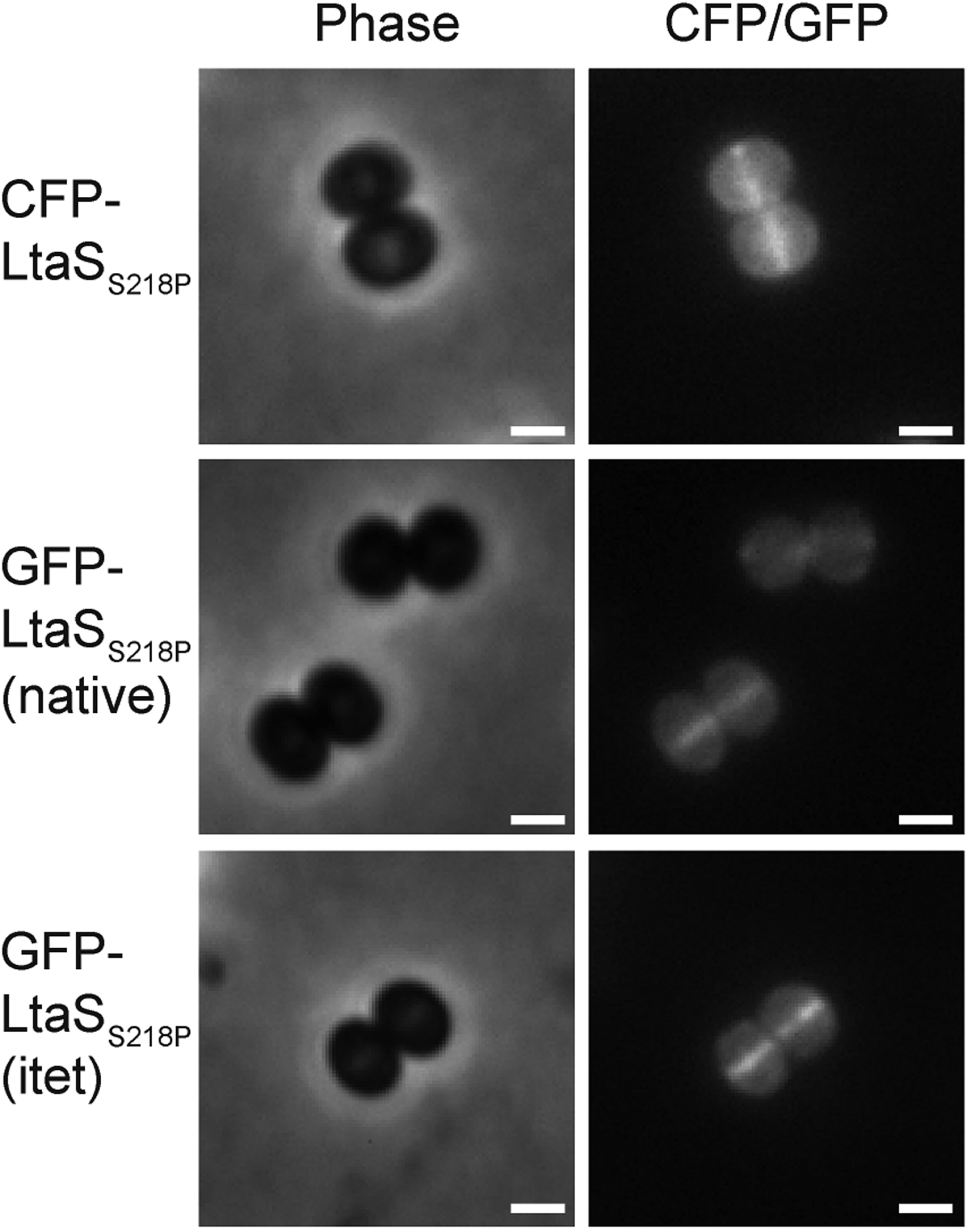
Localization of LtaS in *S. aureus*. *S. aureus* LAC* strains containing pCL55–p*ltaS–cfp–ltaS*_S__218__P_ (CFP–LtaS_S__218__P_), pCL55–p*ltaS–gfp_P7_–ltaS*_S__218__P_ [GFP–LtaS_S__218__P_ (native)] or p*itet–gfp_P7_–ltaS*_S__218__P_ [GFP–LtaS_S__218__P_ (itet) grown in the presence of 200 ng ml^−1^ Atet] were grown to mid-exponential phase, mounted on a 1.2% PBS agarose slide and subsequently observed by fluorescence microscopy. Phase contrast (left) and fluorescence (right) images are shown. Scale bar = 1 μm. A larger field of cells is shown in Fig. S5.

Next, the localization of the LTA synthase enzyme LtaS was determined in the *S. aureus* strain LAC*. Since the wild type LtaS protein is naturally processed in the cell, a less efficiently cleaved LtaS_S218P_ variant was used for these experiments. Initially the localization of the fully functional CFP–LtaS_S218P_ fusion protein was investigated. The fluorescence signal for this fusion was, however, very low and despite the use of the less cleavable LtaS_S218P_ variant, a strong cytoplasmic signal was detected in this strain (Fig. 5). As noted above, this is likely due to some degradation of the fusion protein. The strongest fluorescence signal was detected at the mid-cell and unlike the localization patterns of the YpfP and LtaA fusions, no clearly discernible fluorescence could be detected at the lateral membrane in the majority of cells expressing the CFP–LtaS_S218P_ fusion. This suggested that the LtaS protein localized predominantly to the division site (Figs 5 and S5). Next, the localization pattern of the partially functional GFP_P7_–LtaS_S218P_ expressed in LAC* from the native P*ltaS* or the inducible *itet* promoter in the presence of the Atet was determined. The fluorescence signal from both of these fusion constructs was stronger and the reduced cytoplasmic signal resulted in clearer images, again indicating the accumulation of LtaS at the mid-cell (Figs 5 and S5). FR values of 2.57 ± 0.66 and 2.91 ± 0.83 were obtained for LAC* expressing GFP_P7_–LtaS_S218P_ from the native *ltaS* and the inducible *itet* promoter, respectively (Fig. 4), providing further evidence for the enrichment of the LtaS protein at the division site.

### LTA is not surface exposed in *S. aureus* strains RN4220 and LAC*

**Fig. 6:**
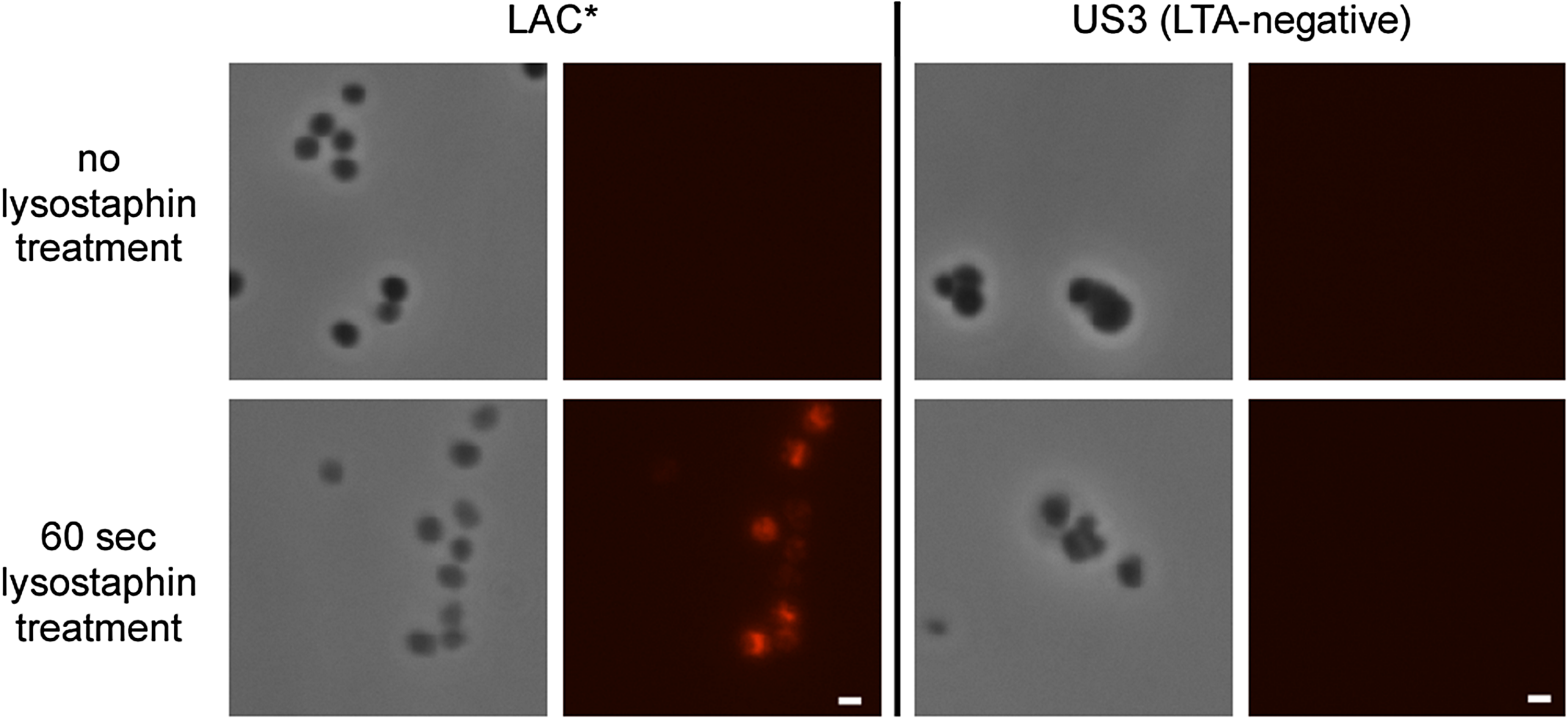
Localization of LTA as assessed by immunofluorescence microscopy. *S. aureus* LAC* and the isogenic LTA negative strain US3 were grown to mid-exponential phase and fixed as described in the experimental procedures section. Where indicated cells were digested with a final concentration of 10 μg ml^−1^ lysostaphin for 60sec. LTA was detected by incubating the cells with a mouse monoclonal polyglycerolphosphate specific primary antibody followed by incubation with anti-mouse Alexafluor 546 conjugated secondary antibody. Samples were subsequently observed by fluorescence microscopy. Scale bar = 1 μm.

Next, to determine the cellular location of the LTA polymer itself in *S. aureus*, an immunofluorescence experiment was performed. To this end, the protein A negative *S. aureus* strain RN4220Δ*spa* and the MRSA strain LAC* as well as previously described isogenic LTA-negative *ltaS* mutant strains 4S5 (RN4220Δ*spa*-derived) and US3 (LAC*-derived) were grown to mid-exponential phase, washed, fixed and mounted on polylysine coated slides. The LTA polymer was subsequently stained using a monoclonal polyglycerolphosphate specific IgG antibody and a fluorescently labelled secondary antibody. Interestingly, no LTA specific signal was detected for any of the strains when the samples were analysed by fluorescence microscopy (Figs 6 and S6). A fluorescence signal was only observed on cells when the peptidoglycan was digested with lysostaphin prior to incubation with the primary antibody. The obtained signal was LTA specific as only samples from the WT strains but not the LTA-negative strains showed a fluorescent signal and identical results were obtained for the RN4220Δ*spa*-derived (Fig. S6) and LAC*-derived (Fig. 6) strains. These data indicate that the LTA polymer is not a cell surface exposed molecule, as often depicted in the literature.

## Discussion

LTA is a cell membrane component found in a variety of Gram-positive bacteria ranging from the Actinobacteria to the Firmicutes (Rahman *et al*., 2009; Reichmann and Gründling, 2011). Gram-positive pathogens with an altered LTA structure, such as mutant strains of *S. aureus* and *Enterococcus faecalis*, demonstrate an increase in the susceptibility to opsonophagocytosis, a decrease in the ability to adhere to and invade host cells and attenuated virulence (Collins *et al*., 2002; Doran *et al*., 2005; Theilacker *et al*., 2009; Sheen *et al*., 2010). Furthermore, depletion of LTA in *S. aureus* leads to aberrant positioning of the septa and eventual cell lysis, highlighting that this polymer is not only important for an interaction with the host, but also for normal cell growth and cell division (Gründling and Schneewind, 2007b). Similar phenotypes have been observed for *L. monocytogenes* and *B. subtilis*, where *ltaS* mutant strains display a filamentation phenotype and in *B. subtilis* this is accompanied by a delocalization of FtsZ, a key cell division protein (Schirner *et al*., 2009; Webb *et al*., 2009).

**Fig. 7:**
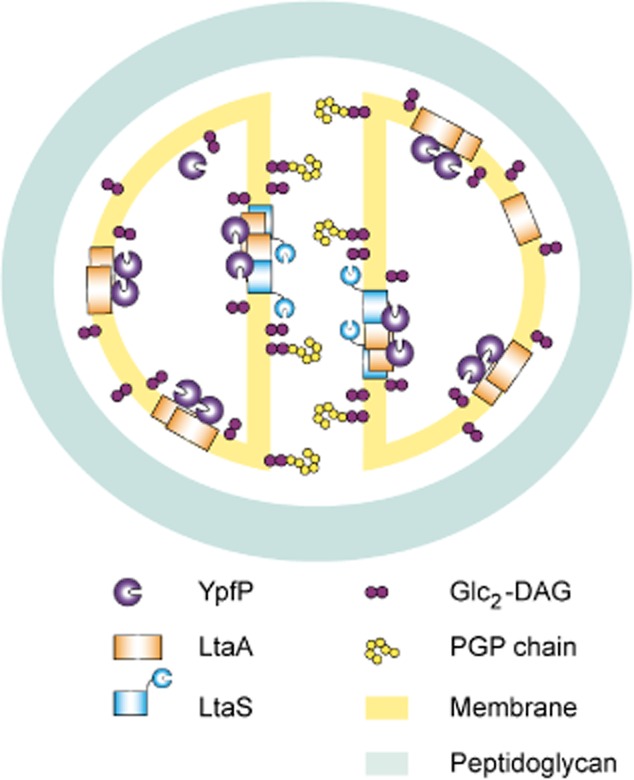
Model for the localization of the LTA synthesis machinery in *S. aureus*. All three *S. aureus* core LTA synthesis proteins, YpfP, LtaA and LtaS self-interact and interact with one another. YpfP and LtaA are distributed throughout the membrane while LtaS accumulates preferentially at the site of cell division. This leads to a model where glycolipids might be synthesized all around the membrane while the LTA backbone production may occur predominantly at the septum prior to cell separation.

In the current study on the *S. aureus* LTA synthesis proteins, data are provided that indicate the formation of a multi-enzyme LTA synthesis complex involving YpfP, LtaA, LtaS and DltD, which further interacts with numerous cell division and peptidoglycan synthesis proteins (Fig. 1). As summarized in the model shown in Fig. 7, the predominant accumulation of LtaS at the septum of *S. aureus*, as observed by microscopy using fluorescent protein fusions (Figs 4, 5 and S5), suggests that new PGP backbone chains of LTA are mainly produced at the division site in this organism. When we attempted to determine the cellular location of the LTA polymer itself by immunofluorescence microscopy and using a polyglycerolphosphate specific antibody, no LTA-specific signal could be detected in intact cells (Figs 6 and S6). Only following digestion of the peptidoglycan with lysostaphin was it possible to detect the polymer. While in several cells the most intense fluorescence signal was obtained at the division site, we do not want to draw firm conclusions regarding the subcellular location of the LTA polymer as the peptidoglycan itself might be more susceptible to lysostaphin treatment at the cell division site. However, these results highlight that in both *S. aureus* strains analysed in this study, the LTA polymer is likely not a surface exposed polymer. Indeed, we have already previously noted (Reichmann and Gründling, 2011) that if the cell wall in *S. aureus* has a thickness of approximately 36 nm as estimated based on cryo-transmission electron microscopy images (Matias and Beveridge, 2006; 2007) and an LTA polymer with 25 repeating units has a length of less than 20 nm when fully extended (Labischinski *et al*., 1991; Fischer, 1994), the polymer may not be able to protrude through the cell wall in *S. aureus* as long as it is linked to the membrane. Our data on *S. aureus* are consistent with EM data presented by Matias and Beveridge for *B. subtilis* and their observation that LTA is a major component of the inner cell wall zone, also called the Gram-positive ‘periplasm’ and not necessarily the outer cell wall zone (Matias and Beveridge, 2008).

In contrast to LtaS, the other two proteins involved in the synthesis of the core LTA structure, YpfP and LtaA, were both found to be distributed all around the membrane (Fig. 3) indicating that glycolipid and hence LTA anchor production might take place throughout the membrane (Fig. 7). Therefore, a full LTA synthesizing complex may be located only at the site of cell division, where it can interact with the cell division machinery, while a second subcomplex consisting of YpfP and LtaA, dedicated to the synthesis and translocation of Glc_2_-DAG, is more evenly distributed throughout the membrane (Fig. 7). This might imply that the Glc_2_-DAG lipid itself has a separate function within the *S. aureus* membrane, which is emphasized by the estimate that 7–8% of the membrane lipids in *S. aureus* are free glycolipids (Koch *et al*., 1984).

YfpP is a predicted cytoplasmic protein, but as one might expect based on its function as the enzyme that transfers UDP-Glc onto the membrane lipid DAG, this protein primarily localizes to the membrane. However, the localization patterns in *S. aureus* differs from that in *B. subtilis*, where the protein is enriched at the cell division site or found at discrete spots in the membrane (Nishibori *et al*., 2005; Weart *et al*., 2007; Chien *et al*., 2012). Especially in *S. aureus* RN4220 the protein appears to be quite uniformly distributed through the cell membrane. Its localization to the membrane is also independent of the substrate UDP-Glc, since its localization pattern did not change in its absence (Figs 3B and S4). If recruited to the membrane via an interaction with cell division or peptidoglycan synthesis proteins, as detected in this study, one would expect to find the protein specifically at the division site. Since this is not the case, it seems more plausible that YpfP is recruited to the membrane perhaps through a direct interaction with DAG, the receptor molecule to which it transfers UDP-Glc, and which constitutes as much as 20% of the lipid bilayer (Koch *et al*., 1984).

The role of the membrane protein LtaA itself is not completely understood, though it has been hypothesized that this protein is involved in the flipping of the glycolipid anchor from the inner to the outer leaflet of the membrane (Gründling and Schneewind, 2007a). This is based on the observation that in an *ltaA* mutant *S. aureus* strain, which produces near wild type levels of Glc_2_-DAG, only a small fraction of the LTA backbone is linked on the outer membrane leaflet via a glycolipid while the majority is anchored by DAG (Gründling and Schneewind, 2007a). The observed protein–protein interactions between LtaA, YpfP and LtaS (Fig. 1) provide further evidence for an involvement of this membrane protein in the LTA synthesis process.

DltD, one of the four proteins required for the incorporation of d-alanines into LTA, showed multiple interactions with the core LTA synthesis enzymes LtaS, YpfP and LtaA suggesting a physical co-ordination between LTA synthesis and its modification (Fig. 1). The function of DltD in the modification process is not understood. We have recently shown that DltD has an N-terminus in, C-terminus out membrane topology (Reichmann *et al*., 2013) and therefore likely functions on the outside of the cell in the final step of the d-alanine modification process, as suggested in the original model proposed by Werner Fischer and colleagues (Perego *et al*., 1995). Consistent with this membrane topology, positive interactions were only detected when the adenylate cyclase domain was fused to the N-terminus of DltD and therefore would remain in cytoplasm of the cell, which is a prerequisite for adenylate cyclase activity. Furthermore, no interactions were detected with the membrane protein DltB. However, it should be noted that based on TMHMM membrane prediction program both the N- and C-terminus of the DltB are likely to be located on the outside of the cell and hence even if interactions with other proteins take place, it would not be possible to detect them with the BACTH system.

A function for LTA as the binding receptor for the major autolysin Atl in *S. aureus* and *Staphylococcus epidermidis* has been proposed (Zoll *et al*., 2012). While it has been known for some time that the repeat domains R1-R2 and R3 within Atl are responsible for a specific targeting of this enzyme to the cell division site (Oshida *et al*., 1995; Baba and Schneewind, 1998), experimental evidence was presented recently indicating a direct interaction between the R1 and R2 repeats and LTA (Zoll *et al*., 2012). Besides *in vitro* binding studies using purified components which provided support for this notion, it was also shown that the binding of fluorescently labelled R1-R2 is abrogated in an *S. aureus* strain lacking LTA (Zoll *et al*., 2012). Based on these data, it was proposed that LTA acts as a receptor for Atl and targets the autolysin during cell separation. This would be consistent with findings in this study, which suggests that the LTA backbone is predominantly produced at the cell division site in *S. aureus*. However it should be noted that even if new LTA is only synthesized at the cell division site, immune EM studies performed many years ago indicate that LTA in *S. aureus* is present throughout the membrane, (Aasjord and Grov, 1980) and based on the immunofluorescence experiments performed in this work, we cannot make reliable conclusions about the cellular localization of the LTA polymer itself. While this study provides experimental evidence for a physical interaction between multiple cell division, peptidoglycan synthesis and LTA synthesis proteins, additional work is clearly needed to understand how these different processes are co-ordinated with one another.

## Experimental procedures

### Bacterial strains and growth conditions

All strains used in this study are listed in Table S1. *E. coli* strains were grown at 37°C or 30°C as indicated in Luria–Bertani (LB) medium supplemented with the following antibiotics, inducers and indicators where appropriate: ampicillin (Amp), 100 μg ml^−1^; kanamycin (Kan), 30–50 μg ml^−1^; spectinomycin (Spec), 100 μg ml^−1^; streptomycin (Strep), 100 μg ml^−1^; tetracycline (Tet), 10 μg ml^−1^; isopropyl β-d-thiogalactosidase (IPTG), 0.5 mM; 5-bromo-4-chloro-3-indolyl-β-D-galactopyranoside (X-gal), 40 μg ml^−1^. *S. aureus* strains were grown at 37°C in tryptic soy broth (TSB), with the addition of the following antibiotics and inducers when necessary: chloramphenicol (Cam), 5–10 μg ml^−1^; erythromycin (Erm), 10 μg ml^−1^; kanamycin (Kan), 90–200 μg ml^−1^; isopropyl β-d-thiogalactosidase (IPTG), 1 mM; anhydrotetracycline (Atet), 200 ng ml^−1^.

### Strain and plasmid construction

Primers used in this study are listed in Table S2. For bacterial adenylate cyclase two hybrid (BACTH) interaction studies, *ypfP*, *ltaA*, *ltaS*, *dltA*, *dltB*, *dltC* and *dltD* were cloned into the BACTH vectors pKT25, pKNT25, pUT18 and pUT18C. For cloning into pKT25, the genes encoding YpfP, LtaA, LtaS, DltA, DltB, DltC and DltD were amplified from *S. aureus* Newman chromosomal DNA with primers ANG 605/606, 601/602, 603/604, 591/592, 593/594, 595/596 and 597/598 respectively. The PCR products and pKT25 were digested with BamHI and KpnI and ligated. The resulting plasmids were initially obtained in *E. coli* XL1 Blue yielding strains ANG1291, ANG1289, ANG1290, ANG1284, ANG1285, ANG1286 and ANG1287.

For construction of pKNT25, pUT18 and pUT18C vectors containing the LTA synthesis genes, all genes were first amplified from *S. aureus* Newman chromosomal DNA with the primers described above and ligated to the TOPO® cloning vector pCR8/GW/TOPO vector (Invitrogen), following the manufacturer's instructions. Plasmids pCR8-*ypfP*, pCR8-*ltaA*, pCR8-*ltaS*, pCR8-*dltA*, pCR8-*dltB*, pCR8-*dltC* and pCR8-*dltD* were recovered in One Shot *E. coli* cells yielding strains ANG1299, ANG1297, ANG1298, ANG1292, ANG1293, ANG1294 and ANG1295. These plasmids were then digested with BamHI and KpnI and the appropriate fragment ligated with pKNT25, pUT18 and pUT18C that had been digested with the same enzymes. The resulting plasmids were recovered in *E. coli* XL1 Blue yielding strains ANG1308, ANG1306, ANG1307, ANG1301, ANG1302, ANG1303 and ANG1304; ANG1318, ANG1316, ANG1317, ANG1311, ANG1312, ANG1313 and ANG1314; ANG1326, ANG1324, ANG1325, ANG1319, ANG1320, ANG1321 and ANG1322.

For construction of pKT25-*zapA* and pUT18C-*zapA*, *zapA* was amplified from *S. aureus* SH1000 chromosomal DNA with primers GLUSH302AJ5′ and GLUSH302AJ3′. The PCR product and vectors were digested with BamHI and EcoRI, ligated and transformed into *E. coli*, yielding plasmids pALB9 and pALB10 respectively.

Vectors for the expression of fluorescent protein fusions with YpfP, LtaA and LtaS were produced as follows: Green fluorescent protein (GFP) followed by a 3× EAAAK amino acid linker region was fused to the N-terminal end of YpfP and expressed under the native *ypfP* promoter from the *S. aureus* chromosomal integration vector pCL55. The *ypfP* promoter sequence was amplified from *S. aureus* Newman chromosomal DNA using primers 1326/1327 and *gfp* was amplified from pBCB1-GE using primers 1328/1329. The resulting PCR products were fused by splice overlap extension (SOE) PCR using primers 1326/1329. The *ypfP* sequence was amplified from *S. aureus* Newman chromosomal DNA using primers 1330/258. Both PCR products were digested with SacII, ligated and the ligation product was used in a PCR reaction with primers 1326/258. This PCR product and plasmid pCL55 were digested with EcoRI and KpnI and ligated. The resulting plasmid pCL55–p*ypfP–gfp–ypfP* was initially recovered in *E. coli* XL1 Blue yielding strain ANG2195. Introduction into *S. aureus* RN4220Δ*spa*, RN4220Δ*spa*Δ*ypfP*, RN4220Δ*spa*Δ*pgcA* by electroporation and subsequent transduction into *S. aureus* strain LAC* yielded strains ANG2199, ANG2202, ANG2587 and ANG2390 respectively.

The integrative plasmid pBCB1-GE was used for the expression of an LtaA–GFP fusion protein. The *ltaA* sequence was amplified from *S. aureus* Newman chromosomal DNA using primers 1324/1325, digested with KpnI and NheI and ligated with pBCB1-GE, which had been digested with the same enzymes. The plasmid pBCB1-GE-*ltaA* was recovered in *E. coli* XL1 Blue resulting in strain ANG2172. Integration of pBCB1-GE-*ltaA* at the native *ltaA* locus in *S. aureus* RN4220Δ*spa* and subsequent transduction into *S. aureus* strain LAC* gave rise to strains ANG2196 and ANG2389, respectively, and resulted in expression of the LtaA–GFP fusion under the native *ltaA* promoter, with an additional copy of *ltaA* under the control of the IPTG-inducible P*spac* promoter. The fast folding GFP variant GFP_P7_ (Fisher and DeLisa, 2008) followed by a 3× EAAAK amino acid linker region was fused to the N-terminus of LtaS_S218P_. This fusion was expressed either from the native *ltaS* promoter or the Atet inducible *itet* promoter. For construction of the GFP_P7_–LtaS_S218P_ fusion under the native promoter control, the *ltaS* promoter sequence was amplified from *S. aureus* Newman chromosomal DNA using primers 1334/1697 and the *gfp_P7_* sequence from pTrc99A–*gfp*_P7_ using primers 1698/1699. The PCR products were fused by SOE PCR using primers 1334/1701 and the resulting product digested with SacII. The *ltaS_S218P_* sequence was amplified from pOK–*ltaS_S218P_* using primers 1700/317 and digested with SacII. These two PCR products were ligated and re-amplified using primers 1334/317. The resulting PCR product and plasmid pCL55 were digested with EcoRI and KpnI and ligated. Plasmid pCL55–p*ltaS*–*gfp*_P7_–*ltaS_S218P_* was initially obtained in *E. coli* XL1 Blue yielding strain ANG2991 and subsequently introduced into *S. aureus* RN4220Δ*spa*, RN4220*iltaS* and LAC* yielding strains ANG3019, ANG3035 and ANG3023 respectively. For construction of the GFP_P7_-LtaS_S128P_ fusion under the control of the Atet inducible *itet* promoter, the *gfp_P7_* sequence was amplified from pTrc99A–*gfp*_P7_ using primers 1702/1701 and the product digested with SacII. The *ltaS_S218P_* sequence was amplified from pOK–*ltaS_S218P_* using primers 1337/319 and digested with SacII. The PCR products were ligated and re-amplified using primers 1702/319. The resulting PCR product and p*itet* were digested with AvrII and BglII and ligated. Plasmid p*itet*–*gfp_P7_*–*ltaS_S218P_* was initially obtained in *E. coli* XL1 Blue yielding strain ANG2993 and subsequently introduced into *S. aureus* strains RN4220Δ*spa*, RN4220*iltaS* and LAC* yielding strains ANG3021, ANG3037 and ANG3024 respectively.

For construction of plasmid pCL55–p*ltaS*–*cfp–ltaS*_S218P_, plasmids pCN34–p*ltaS*–*cfp*–*ltaS_S128P_* and p*itet–cfp–ltaS*_S218P_ were constructed as intermediates. Plasmid pCN34–p*ltaS*–*cfp*–*ltaS_S128P_* was produced by amplifying the *ltaS* promoter region from pCL55–p*ltaS–YFP–*SAV0719 using primers 086/828, the *cfp* sequence from plasmid pS10–CFPopt using primers 829/830 and the *ltaS*_S218P_ sequence from plasmid pOK–*ltaS*_S218P_ using primers 831/087. The resulting products were fused by SOE PCR using primers 086/087, digested with BamHI and SalI and ligated with plasmid pCN34, which had been digested with the same enzymes. Plasmid pCN34–p*ltaS–cfp–ltaS*_S128P_ was obtained in *E. coli* XL1 Blue yielding strain ANG1734. Plasmid p*itet–cfp–ltaS*_S218P_ was produced by amplifying the *cfp–ltaS_S218P_* fragment from plasmid pCN34–p*ltaS–cfp–ltaS*_S218P_ using primers 1112/1115. The resulting PCR product was digested with SalI and SacII and ligated with plasmid p*itet*-*lacZ*, which had been digested with the same enzymes. Plasmid p*itet–cfp–ltaS*_S218P_ was recovered in *E. coli* XL1 Blue yielding strain ANG1825. For construction of plasmid pCL55–p*ltaS–cfp–ltaS*_S218P_, both the pCN34–p*ltaS–cfp–ltaS*_S218P_ (ANG1734) and p*itet–cfp–ltaS*_S218P_ (ANG1825) plasmids were digested with KpnI and NheI. The fragment containing the *ltaS* promoter, *cfp* and the first 647 base pairs of *ltaS*_S218P_ from pCN34–p*ltaS–cfp–ltaS*_S218P_ was ligated to the vector backbone of p*itet–cfp–ltaS*_S218P_ resulting in plasmid pCL55–p*ltaS–cfp–ltaS*_S218P_. This plasmid was recovered in *E. coli* XL1 Blue yielding strain ANG2766 and then introduced by electroporation in *S. aureus* strains RN4220Δ*spa*, RN4220*iltaS* and by transduction into LAC* resulting in strains ANG2833, ANG2834 and ANG2835, respectively.

Plasmid pCL55–p*tet*–*gfpmut2* was used as a control for the expression of GFP from the tetracycline promoter. For its construction, the *gfpmut2* fragment containing a ribosome binding site was amplified with primers 166/167, digested with AvrII and SacII and ligated with vector p*itet* that had been digested with the same enzymes. The resulting plasmid p*itet*–*gfpmut2* was recovered in *E. coli* XL1 Blue, resulting in strain ANG287. Next, the tetracycline promoter *gfp* fragment was amplified with primers 192/184 and the resulting PCR fragment and plasmid pCL55 were digested with BamHI and KpnI and ligated. The resulting plasmid pCL55–*ptet*–*gfpmut2* was recovered in *E. coli* XL1 Blue and subsequently introduced into *S. aureus* RN4220 and LAC* yielding strains ANG302, ANG303 and ANG2397 respectively.

### Bacterial adenylate cyclase two-hybrid screen

pKT25/pKNT25 and pUT18/pUT18C vectors containing the genes of interest were introduced into chemically competent BTH101 cells and transformation reactions spotted onto LB agar supplemented with 100 μg ml^−1^ Amp, 50 μg ml^−1^ Kan, 40 μg ml^−1^ X-gal and 0.5 mM IPTG. Plates were incubated at 30°C for 36–44 h before images were taken and coloration assessed. Experiments were performed in triplicate and a representative result is shown.

### Fluorescence microscopy

*S. aureus* strains were grown overnight at 37°C with shaking in 5 ml TSB supplemented with the appropriate antibiotics. The following day, cultures were diluted 1:1000 into 20 ml TSB and grown to an OD_600_ of 0.8 (mid-exponential phase). Bacteria from a 1 ml culture aliquot were pelleted by centrifugation at 17 000 *g* for 1 min, suspended in 15 μl PBS and 0.5 μl was mounted on microscope slides covered in a thin layer of 1.2% agarose in PBS. Cells were observed using a Zeiss Axio Observer inverted microscope equipped with a Photometrics CoolSNAP HQ2 camera (Roper Scientific) and Metamorph 7.5 software (Molecular Devices).

To quantitatively assess the protein localization and distinguish between membrane and septal protein localization, fluorescence values at the septum and the lateral membrane were determined for 200 cells with fully formed septa. The lateral membrane was identified and highlighted in phase contrast images and the corresponding fluorescence signal was then quantified using Metamorph 7.5 software to obtain the fluorescence intensity value for the lateral wall. A FR value was calculated by dividing the septal fluorescence value by the lateral membrane fluorescence values. Membrane localized proteins are expected to yield an FR value of around 2, while an FR value above 2 indicates preferential septal localization.

### Immunofluorescence microscopy

*S. aureus* strains RN4220Δ*spa* and LAC* and the isogenic LTA negative strains 4S5 and US3 were grown overnight at 37°C with shaking in 5 ml TSB supplemented with the appropriate antibiotics. The next day, the cultures were diluted 1:400 (RN4220Δ*spa*, LAC* and 4S5) or 1:200 (US3) into 30 ml fresh TSB and grown to an OD_600_ of 0.5–0.8 (mid-exponential phase). Bacteria from an 18 ml culture aliquot were pelleted by centrifugation at 4000 *g* for 20 min, suspended in 30 μl PBS and vortexed. The samples were mixed with 3 ml of the fixative agent Histochoice (Electron Microscopy Sciences), and incubated for 15 min at room temperature followed by a 30 min incubation step on ice. Next, the bacteria were pelleted by centrifugation at 17 000 *g* for 1 min, suspended in 3 ml PBS and vortexed for 30 s. The wash steps were repeated three times and after the final wash the bacteria were suspended in 200 to 500 μl 50 mM glucose, 20 mM Tris HCl pH 7.5, 10 mM EDTA (GTE) buffer.

For microscopy analysis, 10-well microscope slides were coated with polylysine by adding 25 μl of a 0.01% polylysine solution to each well and the slide was incubated at room temperature for 5 min. The wells were washed eight times with water and air-dried for 5 min after. Next, 25 μl of the fixed cells were added per well or where indicated 100 μl aliquots of the fixed cells were first mixed with 1 μl of a 1 mg/ml lysostaphin solution and 25 μl of the bacterial suspension was subsequently added to a well. After 60 s. the cells were washed eight times with PBS. Following the last wash step, the wells were briefly air-dried and the cells were subsequently rehydrated by adding 25 μl PBS and incubated at room temperature for 5 min. The PBS solution was replaced with 25 μl blocking solution (2% bovine serum albumin, 50 μg ml^−1^ human IgG in PBS) and incubated for 1 h at room temperature. The blocking solution was removed and 30 μl of a 1:50 dilution of the mouse monoclonal LTA antibody in blocking solution was added and incubated overnight at 4°C. As negative control, cells were instead incubated overnight with 30 μl of blocking solution without antibody. The next day, each well was washed eight times with 25 μl PBS and subsequently incubated for 15 min with blocking solution. The liquid was removed and 30 μl of a 1:500 dilution of an anti-mouse Alexafluor 546 conjugated secondary antibody in blocking solution was added. The slides were incubated at room temperature in the dark for 2 h, and the wells were subsequently washed eight times with PBS. Vectashield mounting medium (Vector Laboratories) was added to each well and the slides covered with a coverslip. The samples were observed using a Zeiss Axio Vert 200 M widefield microscope equipped with a Hamamastu ORCA-ER camera and Volocity software at the FILM Facility at Imperial College London. Experiments were performed in triplicate and representative results are shown.

### *S. aureus* growth curves and detection of LTA by Western blotting

*S. aureus* growth curves of the inducible *ltaS* strains and LTA detection by Western blot were performed as previously described, with the exception that data points for growth curves were plotted starting at the 4 h time point, at which point all cultures were back-diluted (Wörmann *et al*., 2011a).

### Detection of fluorescent protein fusions by Western blotting or fluorescence imaging

*S. aureus* strains were grown overnight at 37°C in 5 ml TSB supplemented with the appropriate antibiotics. The next day, the cultures were back-diluted 1:100 into 5 ml TSB medium and after 4 h of growth at 37°C, bacteria from a 1 ml culture aliquot were pelleted by centrifugation at 17 000 *g* for 5 min. The pellet was suspended in 100 mM Tris pH 7.5, 10 mM MgCl_2_, 0.5 M sucrose (TSM) buffer containing 200 μg ml^−1^ lysostaphin and 100 μg ml^−1^ DNase normalized based on the OD_600_ reading of the culture, that is a culture with an OD_600_ of 1 was suspended in 15 μl buffer. The samples were incubated at 37°C for 30 min and subsequently an equal volume of 2× protein sample buffer was added. All samples were centrifuged at 17 000 *g* for 5 min and 10 μl separated on a 10% polyacrylamide gel. A mouse monoclonal GFP antibody (Invitrogen) and an HRP-conjugated anti-mouse IgG antibody (NEB) were used at 1:4000 and 1:10000 dilutions for Western blot analysis. Samples for the detection of fluorescent proteins using a fluorescence imager were prepared as described above except that cells were lysed in 100 mM Tris-HCl pH 7.5, 10 mM MgCl_2_ buffer containing 200 μg ml^−1^ lysostaphin and 100 μg ml^−1^ DNase. After separating the proteins on a 10% gel, images were taken using a Typhoon FLA 700 imager (GE Healthcare) with a 473 nm filter. Experiments were performed in triplicate and a representative result is shown.

### Phosphoglucomutase activity assay

*S. aureus* strains RN4220Δ*spa* and RN4220Δ*spa*Δ*pgcA* were grown in 250 ml TSB to an OD_600_ of 0.7. Cells were harvested by centrifugation, washed once with 5 ml ice-cold ddH_2_O and suspended in 800 μl ice-cold TEA buffer [100 mM triethanolamine (TEA), 5 mM MgCl_2_ and 2.2 mM EDTA]. Samples were mixed with 0.5 ml volume of 0.1 mm glass beads and lysed in a Fast-Prep machine 3× for 45 s at setting 6 (MP Biomedicals, LLC). Glass beads and unbroken cells were sedimented by centrifugation at 17 000 *g* for 30 min at 4°C. The supernatant fraction was transferred to a fresh tube and the protein concentration in these cytoplasmic extracts determined using the BCA protein assay kit (Pierce). Phosphoglucomutase activity was determined as described in (Lazarevic *et al*., 2005). Briefly, phosphoglucomutase activity was assessed by measuring the conversion rate of α-glucose 1-phosphate to glucose 6-phosphate and subsequent conversion of glucose 6-phosphate to D-Glucono-1,5-lactone 6-phosphate by a glucose 6-phosphate dehydrogenase. The concomitant production of NADPH from NADP was measured spectrophotometrically and quantified using an NADPH standard curve. The reactions were set up in a volume of 1 ml and contained 0.1 mg cell extracts, 0.6 mM NADP (Sigma), 1 U glucose 6-phosphate dehydrogenase (Sigma) and 2 μM α-d-glucose 1,6-diphosphate (Sigma) in TEA buffer. The reactions were initiated by the addition of 1.5 μM final concentration α-d-glucose 1-phosphate and the formation of NADPH over time was monitored spectrophotometrically at 340 nm. The experiment was performed four times and the average values and standard deviations plotted.
