# Supplementary material for: Differential localization of LTA synthesis proteins and their interaction with the cell division machinery in Staphylococcus aureus
Source: Mol Microbiol. 2014 Mar 20;92(2):273–86. doi: 10.1111/mmi.12551 (PMC4065355; doi:10.1111/mmi.12551)
Supplement: Supplementary file 1 [file mmi0092-0273-SD1.pdf]

Supporting information

Supporting Tables

Table S1: Bacterial strains used in this study

Strain	Relevant Features	Reference
	***Escherichia coli* strains**	
XL1 Blue	Cloning strain; TetR - ANG127	Stratagene
One Shot	Cloning strain - ANG1409	Invitrogen
BTH101	BACTH Δ*cya* strain; StrepR - ANG1309	Euromedex
201	pCN34 in DH5α; *E. coli*/*S. aureus* shuttle vector; KanR, AmpR	(Charpentier *et al*., 2004)
ANG243	pCL55 in XL1 Blue; *S. aureus* single-site integration vector; AmpR	(Lee *et al*., 1991)
ANG284	p*itet* in XL1 Blue; pCL55 containing Atet inducible promoter; AmpR	(Gründling & Schneewind, 2007a)
ANG286	p*itet-lacZ* in XL1 Blue; AmpR	Lab strain collection
ANG287	XL1 Blue p*itet*-gfpmut2	This study
ANG302	pCL55-*ptet-gfpmut2* in XL1 Blue; AmpR	This study
ANG1162	pCL55-p*ltaS*-YFP-SAV0719 in XL1 Blue; AmpR	Lab strain collection
ANG1212	p*itet-1TM-eltaS* in XL1 Blue; AmpR	(Wörmann *et al*., 2011b)
ANG1213	p*itet-3TM-eltaS* in XL1 Blue; AmpR	(Wörmann *et al*., 2011b)
ANG1242	pOK-$ltaS_{S218P}$ in XL1 Blue; KanR	(Wörmann *et al*., 2011b)
1265	pKT25 in XL1 Blue; BACTH vector containing an IPTG inducible promoter, T25 and multiple cloning site; KanR	(Karimova *et al*., 2001)
1266	pKNT25 in XL1 Blue; BACTH vector containing an IPTG inducible promoter, multiple cloning site and T25; KanR	(Karimova *et al*., 2005)
1267	pUT18 in XL1 Blue; BACTH vector containing an IPTG inducible promoter, multiple cloning site and T18; AmpR	(Karimova *et al*., 2001)
1268	pUT18C in XL1 Blue; BACTH vector containing an IPTG inducible promoter, T18 and multiple cloning site; AmpR	(Karimova *et al*., 2001)
1269	pKT25-*zip* in XL1 Blue; T25 fused to N-terminus of the leucine zipper of GCN4; KanR	(Karimova *et al*., 1998)
1270	pUT18C-*zip* in XL1 Blue; T18 fused to N-terminus of the leucine zipper of GCN4; AmpR	(Karimova *et al*., 1998)
ANG1284	pKT25-*dltA* in XL1 Blue; T25 fused to N-terminus of DltA; KanR	This study
ANG1285	pKT25-*dltB* in XL1 Blue; T25 fused to N-terminus of DltB; KanR	This study
ANG1286	pKT25-*dltC* in XL1 Blue; T25 fused to N-terminus of DltC; KanR	This study
ANG1287	pKT25-*dltD* in XL1 Blue; T25 fused to N-terminus of DltD; KanR	This study
ANG1289	pKT25-*ltaA* in XL1 Blue; T25 fused to N-terminus of LtaA; KanR	This study
ANG1290	pKT25-*ltaS* in XL1 Blue; T25 fused to N-terminus of LtaS; KanR	This study
ANG1291	pKT25-*ypfP* in XL1 Blue; T25 fused to N-terminus of YpfP; KanR	This study
-	pCR8/GW/TOPO in One Shot *E. coli*; SpecR	Invitrogen
ANG1292	pCR8-*dltA* in XL1 Blue; DltA expressed from a high-copy plasmid; SpecR	This study
ANG1293	pCR8-*dltB* in XL1 Blue; DltB expressed from a high-copy plasmid; SpecR	This study
ANG1294	pCR8-*dltC* in XL1 Blue; DltC expressed from a high-copy plasmid; SpecR	This study
ANG1295	pCR8-*dltD* in XL1 Blue; DltD expressed from a high-copy plasmid; SpecR	This study
ANG1297	pCR8-*ltaA* in XL1 Blue; LtaA expressed from a high-copy plasmid; SpecR	This study
ANG1298	pCR8-*ltaS* in XL1 Blue; LtaS expressed from a high-copy plasmid; SpecR	This study
ANG1299	pCR8-*ypfP* in XL1 Blue; YpfP expressed from a high-copy plasmid; SpecR	This study
ANG1301	pKNT25-*dltA* in XL1 Blue; T25 fused to C-terminus of DltA; KanR	This study
ANG1302	pKNT25-*dltB* in XL1 Blue; T25 fused to C-terminus of DltB; KanR	This study
ANG1303	pKNT25-*dltC* in XL1 Blue; T25 fused to C-terminus of DltC; KanR	This study
ANG1304	pKNT25-*dltD* in XL1 Blue; T25 fused to C-terminus of DltD; KanR	This study
ANG1306	pKNT25-*ltaA* in XL1 Blue; T25 fused to C-terminus of LtaA; KanR	This study
ANG1307	pKNT25-*ltaS* in XL1 Blue; T25 fused to C-terminus of LtaS; KanR	This study
ANG1308	pKNT25-*ypfP* in XL1 Blue; T25 fused to C-terminus of YpfP; KanR	This study
ANG1311	pUT18-*dltA* in XL1 Blue; T18 fused to C-terminus of DltA; AmpR	This study
ANG1312	pUT18-*dltB* in XL1 Blue; T18 fused to C-terminus of DltB; AmpR	This study
ANG1313	pUT18-*dltC* in XL1 Blue; T18 fused to C-terminus of DltC; AmpR	This study
ANG1314	pUT18-*dltD* in XL1 Blue; T18 fused to C-terminus of DltD; AmpR	This study
ANG1316	pUT18-*ltaA* in XL1 Blue; T18 fused to C-terminus of LtaA; AmpR	This study
ANG1317	pUT18-*ltaS* in XL1 Blue; T18 fused to C-terminus of LtaS; AmpR	This study
ANG1318	pUT18-*ypfP* in XL1 Blue; T18 fused to C-terminus of YpfP; AmpR	This study
ANG1319	pUT18C-*dltA* in XL1 Blue; T18 fused to N-terminus of DltA; AmpR	This study
ANG1320	pUT18C-*dltB* in XL1 Blue; T18 fused to N-terminus of DltB; AmpR	This study
ANG1321	pUT18C-*dltC* in XL1 Blue; T18 fused to N-terminus of DltC; AmpR	This study
ANG1322	pUT18C-*dltD* in XL1 Blue; T18 fused to N-terminus of DltD; AmpR	This study
ANG1324	pUT18C-*ltaA* in XL1 Blue; T18 fused to N-terminus of LtaA; AmpR	This study

ANG1325	pUT18C-*ltaS* in XL1 Blue; T18 fused to N-terminus of LtaS; AmpR	This study
ANG1326	pUT18C-*ypfP* in XL1 Blue; T18 fused to N-terminus of YpfP; AmpR	This study
-	pS10-CFPopt in XL1 Blue;	(Sastalla *et al*., 2009)
ANG1734	pCN34-p*ltaS-cfp-ltaS*$_{S218P}$ in XL1 Blue; CFP fused to *ltaS*$_{S218P}$ variant under *ltaS* promoter control; KanR, AmpR	This study
ANG1825	p*itet-cfp-ltaS*$_{S218P}$ in XL1 Blue; CFP fused to *ltaS*$_{S218P}$ variant under Atet inducible promoter; AmpR	This study
2144	pBCB1-GE in XL1 Blue; *S. aureus* integrative vector for GFP fusions; ErmR, AmpR	(Pereira *et al*., 2010)
ANG2172	pBCB1-GE-*ltaA* in XL1 Blue; GFP fused to *ltaA*; ErmR, AmpR	This study
ANG2195	pCL55-p*ypfP-gfp-ypfP* in XL1 Blue; GFP fused to *ypfP* under *ypfP* promoter control; AmpR	This study
2354	pVF30 in XL1 Blue; T25 fused to C-terminus of EzrA; KanR	(Steele *et al*., 2011)
2355	pVF29 in XL1 Blue; T25 fused to C-terminus of FtsZ; KanR	(Steele *et al*., 2011)
2356	pGL540 in XL1 Blue; T25 fused to N-terminus of DivIB; KanR	(Steele *et al*., 2011)
2357	pGL551 in XL1 Blue; T25 fused to N-terminus of DivIC; KanR	(Steele *et al*., 2011)
2358	pGL541 in XL1 Blue; T25 fused to N-terminus of FtsA; KanR	(Steele *et al*., 2011)
2359	pGL542 in XL1 Blue; T25 fused to N-terminus of FtsL; KanR	(Steele *et al*., 2011)
2360	pALB3 in XL1 Blue; T25 fused to N-terminus of FtsW; KanR	(Steele *et al*., 2011)
2361	pGL557 in XL1 Blue; T25 fused to N-terminus of GpsB; KanR	(Steele *et al*., 2011)
2365	pGL550 in XL1 Blue; T25 fused to N-terminus of PBP1; KanR	(Steele *et al*., 2011)
2366	pGL543 in XL1 Blue; T25 fused to N-terminus of PBP2; KanR	(Steele *et al*., 2011)
2367	pGL556 in XL1 Blue; T25 fused to N-terminus of PBP3; KanR	(Steele *et al*., 2011)
2368	pALB8 in XL1 Blue; T25 fused to N-terminus of RodA; KanR	(Steele *et al*., 2011)
2369	pGL559 in XL1 Blue; T25 fused to N-terminus of SepF; KanR	(Steele *et al*., 2011)
2371	pALB9 in XL1 Blue; T25 fused to N-terminus of ZapA; KanR	This study
2373	pVF32 in XL1 Blue; T18 fused to C-terminus of EzrA; AmpR	(Steele *et al*., 2011)
2374	pVF31 in XL1 Blue; T18 fused to C-terminus of FtsZ; AmpR	(Steele *et al*., 2011)
2375	pGL544 in XL1 Blue; T18 fused to N-terminus of DivIB; AmpR	(Steele *et al*., 2011)
2376	pGL564 in XL1 Blue; T18 fused to N-terminus of DivIC; AmpR	(Steele *et al*., 2011)
2377	pGL545 in XL1 Blue; T18 fused to N-terminus of FtsA; AmpR	(Steele *et al*., 2011)
2378	pGL546 in XL1 Blue; T18 fused to N-terminus of FtsL; AmpR	(Steele *et al*., 2011)
2379	pALB6 in XL1 Blue; T18 fused to N-terminus of FtsW; AmpR	(Steele *et al*., 2011)
2380	pGL570 in XL1 Blue; T18 fused to N-terminus of GpsB; AmpR	(Steele *et al*., 2011)
2382	pGL547 in XL1 Blue; T18 fused to N-terminus of PBP2; AmpR	(Steele *et al*., 2011)
2383	pALB14 in XL1 Blue; T18 fused to N-terminus of RodA; AmpR	(Steele *et al*., 2011)
2384	pGL572 in XL1 Blue; T18 fused to N-terminus of SepF; AmpR	(Steele *et al*., 2011)
2386	pALB10 in XL1 Blue; T18 fused to N-terminus of ZapA; AmpR	This study
2555	p25PBP4 in DH5α; T25 fused to C-terminus of PBP4; KanR	(Reed *et al*., 2011)
ANG2766	pCL55-p*ltaS-cfp-ltaS*$_{S218P}$ in XL1 Blue; CFP fused to *ltaS*$_{S218P}$ variant under *ltaS* promoter control; AmpR	This study
2956	pTric99A-*gfp*P7 in DH5α; AmpR	(Fisher & DeLisa, 2008)
ANG2991	pCL55-p*ltaS-gfp*$_{P7}$-*ltaS*$_{S218P}$ in XL1 Blue; AmpR	This study
ANG2993	p*itet-gfp*$_{P7}$-*ltaS*$_{S218P}$ in XL1 Blue; AmpR	This study
	***Staphylococcus aureus* strains**	
SEJ1	RN4220Δ*spa* - ANG314	(Gründling & Schneewind, 2007a)
Newman	Human clinical isolate – ANG112	(Duthie & Lorenz, 1952)
AH1263	LAC*; Erm sensitive derivative of epidemic U.S. CA-MRSA USA300 strain - ANG1575	(Boles *et al*., 2010)
ANG303	pCL55-*ptet-gfpmut2* integrated in strain RN4220 (ANG113); CamR	This study
ANG359	RN4220Δ*spa*Δ*ltaA*; ErmR	(Gründling & Schneewind, 2007a)
ANG370	RN4220Δ*spa*Δ*ypfP*; ErmR	(Gründling & Schneewind, 2007a)
ANG371	RN4220Δ*spa*Δ*pgcA*; ErmR	(Gründling & Schneewind, 2007a)
ANG499	RN4220*iltaS*; strain with IPTG-inducible *ltaS* expression; ErmR, IPTG	(Gründling & Schneewind, 2007b)
ANG586	pCL55 integrated in strain ANG499; CamR, ErmR, IPTG	Lab strain collection
ANG1786	4S5 (RN4220Δ*spa*Δ*ltaS* suppressor strain)	(Corrigan *et al*., 2011)
ANG1827	p*itet* integrated in strain RN4220Δ*spa*Δ*ypfP*; CamR, ErmR	This study
ANG2196	pBCB1-GE-*ltaA* integrated in strain RN4220Δ*spa*; ErmR	This study
ANG2199	pCL55-p*ypfP-gfp-ypfP* integrated in strain RN4220Δ*spa*; CamR	This study
ANG2202	pCL55-p*ypfP-gfp-ypfP* integrated in strain RN4220Δ*spa*Δ*ypfP*; CamR	This study
ANG2389	pBCB1-GE-*ltaA* integrated in strain LAC*; ErmR	This study
ANG2390	pCL55-p*ypfP-gfp-ypfP* integrated in strain LAC*; CamR	This study
ANG2397	pCL55-p*tet-gfpmut2* integrated in strain LAC*; CamR	This study
ANG2398	p*itet* integrated in strains LAC*; CamR	This study
ANG2434	US3 (LAC*Δ*ltaS* suppressor strain pass 4)	(Corrigan *et al*., 2011)
ANG2587	pCL55-p*ypfP-gfp-ypfP* integrated in strain RN4220Δ*spa*Δ*pgcA*; CamR, ErmR	This study
ANG2833	pCL55-p*ltaS-cfp-ltaS*$_{S218P}$ integrated in strain RN4220Δ*spa*; CamR	This study

ANG2834	pCL55-p*ltaS-cfp-ltaS*$_{\mathrm{S218P}}$ integrated in strain RN4220*iltaS*; CamR	This study
ANG2835	pCL55-p*ltaS-cfp-ltaS*$_{\mathrm{S218P}}$ integrated in strain LAC*; CamR	This study
ANG3019	pCL55-p*ltaS-gfp*$_{\mathrm{P7}}$-*ltaS*$_{S218P}$ integrated in strain RN4220Δ*spa*; CamR	This study
ANG3021	p*itet-gfp*$_{\mathrm{P7}}$-*ltaS*$_{S218P}$ integrated in strain RN4220Δ*spa*; CamR	This study
ANG3023	pCL55-p*ltaS-gfp*$_{\mathrm{P7}}$-*ltaS*$_{S218P}$ integrated in strain LAC*; CamR	This study
ANG3025	p*itet-gfp*$_{\mathrm{P7}}$-*ltaS*$_{S218P}$ integrated in strain LAC*; CamR	This study
ANG3035	pCL55-p*ltaS-gfp*$_{\mathrm{P7}}$-*ltaS*$_{S218P}$ integrated in strain RN4220*iltaS*; CamR, ErmR, IPTG	This study
ANG3037	p*itet-gfp*$_{\mathrm{P7}}$-*ltaS*$_{S218P}$ integrated in strain RN4220*iltaS*; CamR, ErmR, IPTG	This study

Table S2: Primers used in this study

Number	Primer name	Sequence
ANG086	5'-BamHI +P SAV0719	CGGGATCCGGAATAGAATATAGAATGCAATTAGAAATG
ANG087	3-SalI SAV0719	ACGCGTCGACCCGAGTTCGTGTTTAAATATTATTTTTTAG
ANG166	5-AvrII-GFP	CCGCCTAGGGGATCCTAAGGAGGAAAAAAAATGAG
ANG167	3-SacII-GFP	TCCCCGCGGTTATTTGTATAGTTCATCCATGCCATGTG
ANG184	5-BamHI-TET	CGGGATCCCCTCGAGTTCATGAAAAAC
ANG192	3-KpnI GFP with STOP	GGGGTACCTTATTTGTATAGTTCATCCATGCCATGTG
ANG258	3-KpnI-SAV1017	GGGGTACCTTATTTAACGAAGAATCTTGCATATAAAGGAACC
ANG317	3-KpnI-SAV719	GGGGTACCCCGAGTTCGTGTTTAAATATTATTTTTTAG
ANG319	3-BglII-SAV719	GAAGATCTCCGAGTTCGTGTTTAAATATTATTTTTTAG
ANG591	5'-BamHI-DltA	CGGGATCCCACAGATATTATTAACAAGCTGCAAGC
ANG592	3'-KpnI-DltA	GGGGTACCCGTCCGTTAATTACCTCTGCAATTTTCTTTC
ANG593	5'-BamHI-DltB	CGGGATCCCATTCCATATGGTGATTTTACATTCTTC
ANG594	3'-KpnI-DltB	GGGGTACCCGTATAAGTTTACCTGAGAAGATTAAAAAG
ANG595	5'-BamHI-DltC	CGGGATCCCGAATTTAGAGAACAAGTATTAAATTTATTAG
ANG596	3'-KpnI-DltC	GGGGTACCCGTCGTAACTCTTCTAATGCTTCAACG
ANG597	5'-BamHI-DltD	CGGGATCCCAAATTAAAACCTTTTTTACCCATTTTAATTAG
ANG598	3'-KpnI-DltD	GGGGTACCCGATTTTTAGGTTTATCTACTTCAGGTTG
ANG601	5'-BamHI-LtaA	CGGGATCCCCAAGATTCTTCGTTAAATAATTACGC
ANG602	3'-KpnI-LtaA	GGGGTACCCGCTTAGCTTTTTCTCTATTTACTATAAAG
ANG603	5'-BamHI-LtaS	CGGGATCCCAGTTCACAAAAAAAAGAAAATTAGTC
ANG604	3'-KpnI-LtaS	GGGGTACCCGTTTTTTAGAGTTTGCTTTAGGTCCTG
ANG605	5'-BamHI-YpfP	CGGGATCCCGTTACTCAAAATAAAAAGATATTGATTATTAC
ANG606	3'-KpnI-YpfP	GGGGTACCCGTTTAACGAAGAATCTTGCATATAAAGG
ANG828	R-3'pltaS-5'CFPopt	cttttgaaaccatGATTCTTTCCCCCGTTATTTAGATAATAAATC
ANG829	F-3'pltaS-5'CFPopt	GGGGAAAGAATCatggtttcaaaaggagaagaattatttac
ANG830	R-5'ltaS-3'CFP	TTTTTGTGAACTcttatataattcatccattccgtgtg
ANG831	F-3'CFPopt-5'ltaS	gaattatataagAGTTCACAAAAAAAAGAAAATTAGTCTTTTTGC
ANG1112	SalI-CFPopt for	TGCGGTCGACGTTTCAAAAGGAGAAGAATTATTTAC
ANG1115	ltaS-TAA-SacII rev	GGGCCGCGGTTATTTTTTAGAGTTTGCTTTAGG
ANG1324	5'-KpnI-ltaA for	CGGGGTACCATGCAAGATTCTTCGTTAAATAATTAC
ANG1325	ltaA-linker NheI-3' rev	CCTAGCTAGCTTTTGCGGCAGCTTCTTTAGCCGCGGCTTCTTTCGCAGCTGCTTCCTTAGCTTTTTCTCTATTTACTATAAAG
ANG1326	5'-EcoRI-pypfP for	CCGGAATTCGCTCCTTTTTCTACAATATGTTTATTATAC
ANG1327	pyfpP-GFP(pBCB) rev	CTTTGCTAGCCATTAATAGCCACCCTCCGTTAGTTTG
ANG1328	pyfpP-GFP(pBCB) for	GGGTGGCTATTAATGGCTAGCAAAGGAGAAGAACTTTTC
ANG1329	GFP(pBCB)-linker-SacII rev	TCCCCGCGGCTTCTTTCGCAGCTGCTTCTTTGTAGAGCTCATCCATGCCATG
ANG1330	SacII-linker-ypfP for	TCCCCGCGGCTAAAGAAGCTGCCGCAAAAGTTACTCAAAATAAAAAGATATTG
ANG1334	5'-EcoRI-pltaS for	CCGGAATTCGGAATAGAATATAGAATGCAATTAG
ANG1337	SacII-linker-LtaS for	GGCCGCGGCTAAAGAAGCTGCCGCAAAAAGTTCACAAAAAAAAGAAAATTAGTC
ANG1697	3-pltaS GFPmut2	TCCTTTACTCATGATTCTTTCCCCCGTTATTTAG
ANG1698	5-pltaS GFPmut2	GGGGAAAGAATCATGAGTAAAGGAGAAGAACTTTTCACTGG
ANG1699	3-GFPmut2-LtaS	TTTTTGTGAACTTTTGTATAGTTCATCCATGCCATGTG
ANG1700	5-GFPmut2-LtaS	GAACTATACAAAAGTTCACAAAAAAAAGAAAATTAGTCTTTTTGC
ANG1701	3-GFPmut2-linker-SacII	TCCCCGCGGCTTCTTTCGCAGCTGCTTCTTTGTATAGTTCATCCATGCCATGTGTAATC
ANG1702	5-AvrII-LtaS rbs-GFPmut2	CCGCCTAGGCTAAATAACGGGGGAAAGAATCATGAGTAAAGGAGAAGAACTTTTCACTGG
	GLUSH302AJ5'	ATAATAGGATCCTATGGCACAGTTTAAAAACAAG
	GLUSH302AJ3'	ATAATAGAATTCTTATTGCTCACGCTGCTG

Supporting Figures

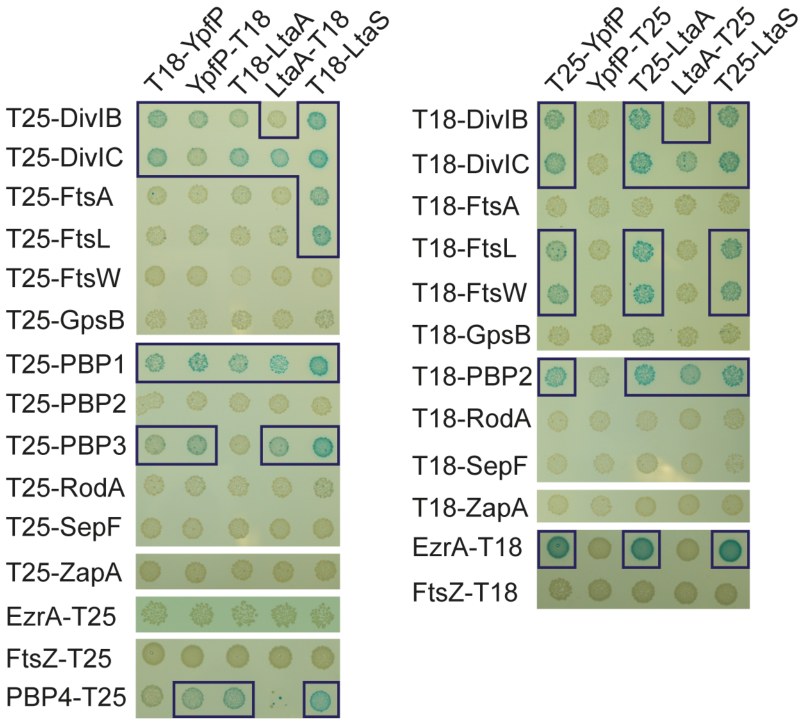

Fig. S1. Qualitative analysis of protein-protein interactions between the core *S. aureus* LTA synthesis proteins, cell division and peptidoglycan synthesis proteins. Protein-protein interactions between the *S. aureus* core LTA synthesis proteins, YpfP, LtaA and LtaS, and the denoted cell division or peptidoglycan synthesis proteins were tested using the BACTH system. *E. coli* BTH101 cells were co-transformed with plasmids expressing the indicated T25- and T18-fusion proteins and spotted onto IPTG and X-gal containing plates. The plates were incubated for 36-44 h at 30ºC and images taken. Transformation reactions yielding blue colonies (further highlighted by a blue box) indicate a positive interaction.

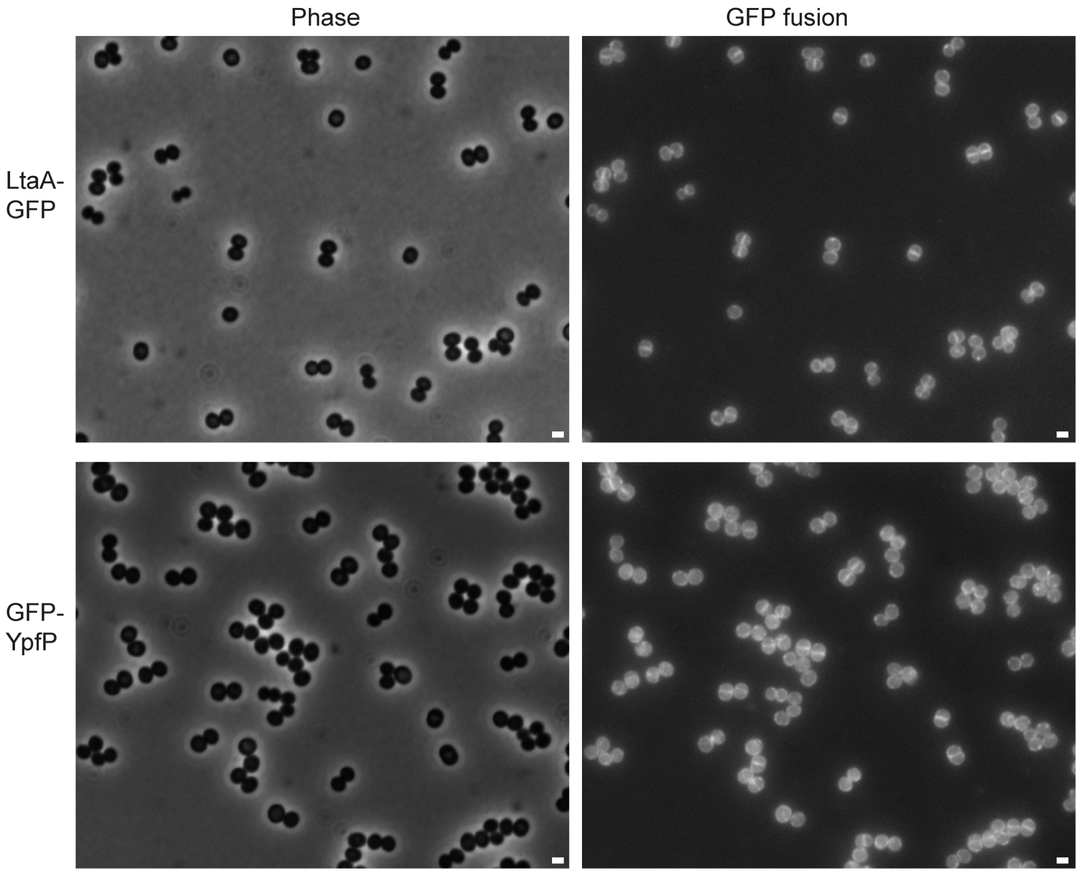

Fig. S2. Localization of YpfP and LtaA in *S. aureus*. *S. aureus* strains LAC* pBCB1-GE-*ltaA* (LtaA-GFP) and LAC* pCL55-p*ypfP-gfp-ypf* (GFP-YpfP) were grown to mid-exponential phase, mounted on a 1.2% PBS agarose slide and subsequently observed by fluorescence microscopy. Phase contrast (left) and fluorescence (right) images are shown. Scale bar = 1 μm.

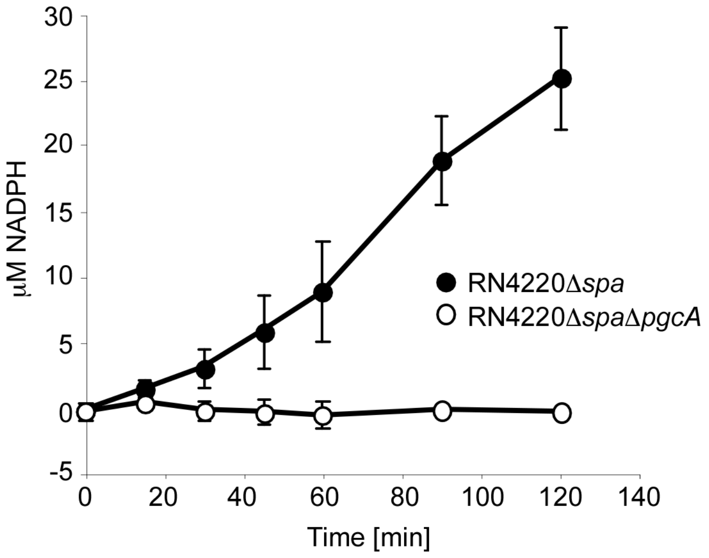

Fig. S3. Phosphoglucomutase activity in wild type and *pgcA* mutant *S. aureus* strains. Cell extracts were prepared from wild type and the *pgcA* mutant *S. aureus* strain. The phosphoglucomutase activity in extracts was assessed in a coupled enzyme assay resulting in the production of NADPH. The assay was performed as described in the material and method section and repeated four times. The average values and standard deviation of the NADPH amount produced over time are plotted for wild type (filled circles) or the *pgcA* mutant (open circles) extracts.

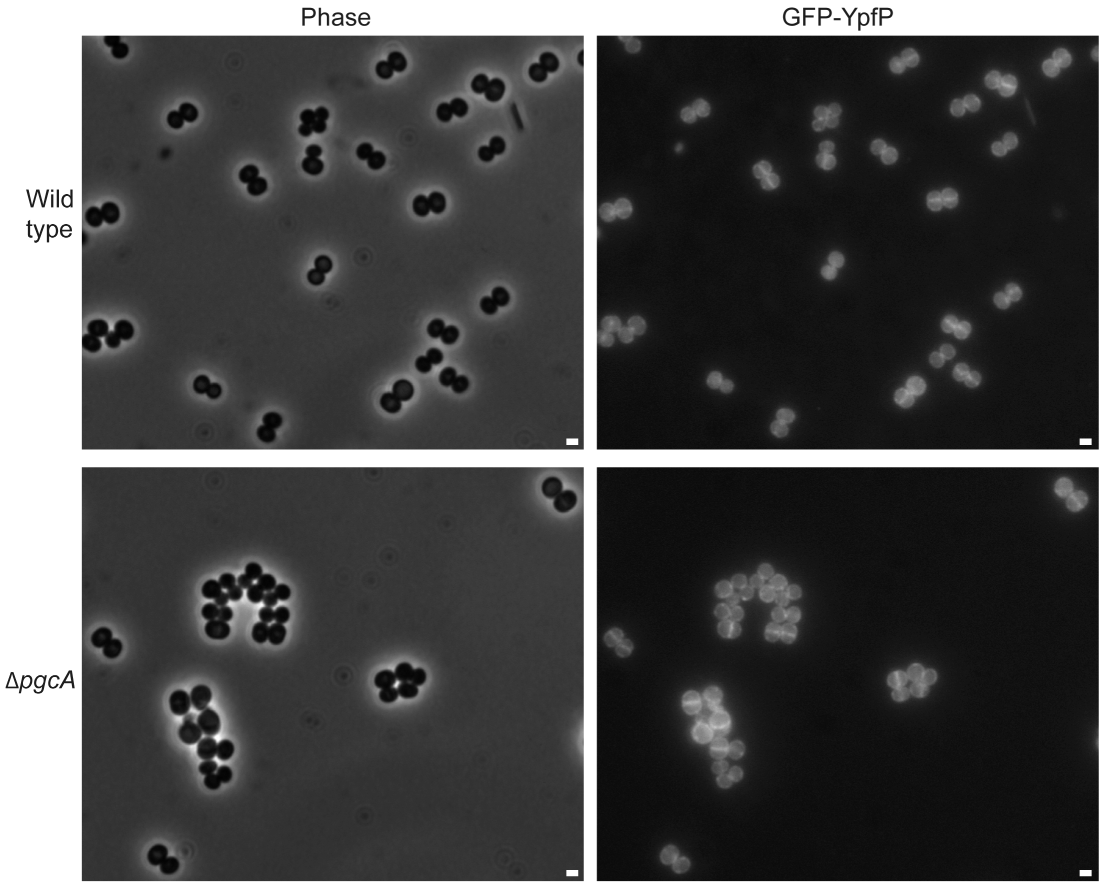

Fig. S4. Localization of YpfP in wild-type and *pgcA* mutant *S. aureus* strains. *S. aureus* strains RN4220Δ*spa* pCL55-p*ypfP-gfp-ypfP* (wild type) and RN4220Δ*spa*Δ*pgcA* pCL55-p*ypfP-gfp-ypfP* (Δ*pgcA*) expressing the GFP-YpfP fusion were grown to mid-exponential phase, mounted on a 1.2% PBS agarose slide and subsequently observed by fluorescence microscopy. Phase contrast (left) and fluorescence (right) images are shown. Scale bar = 1 μm.

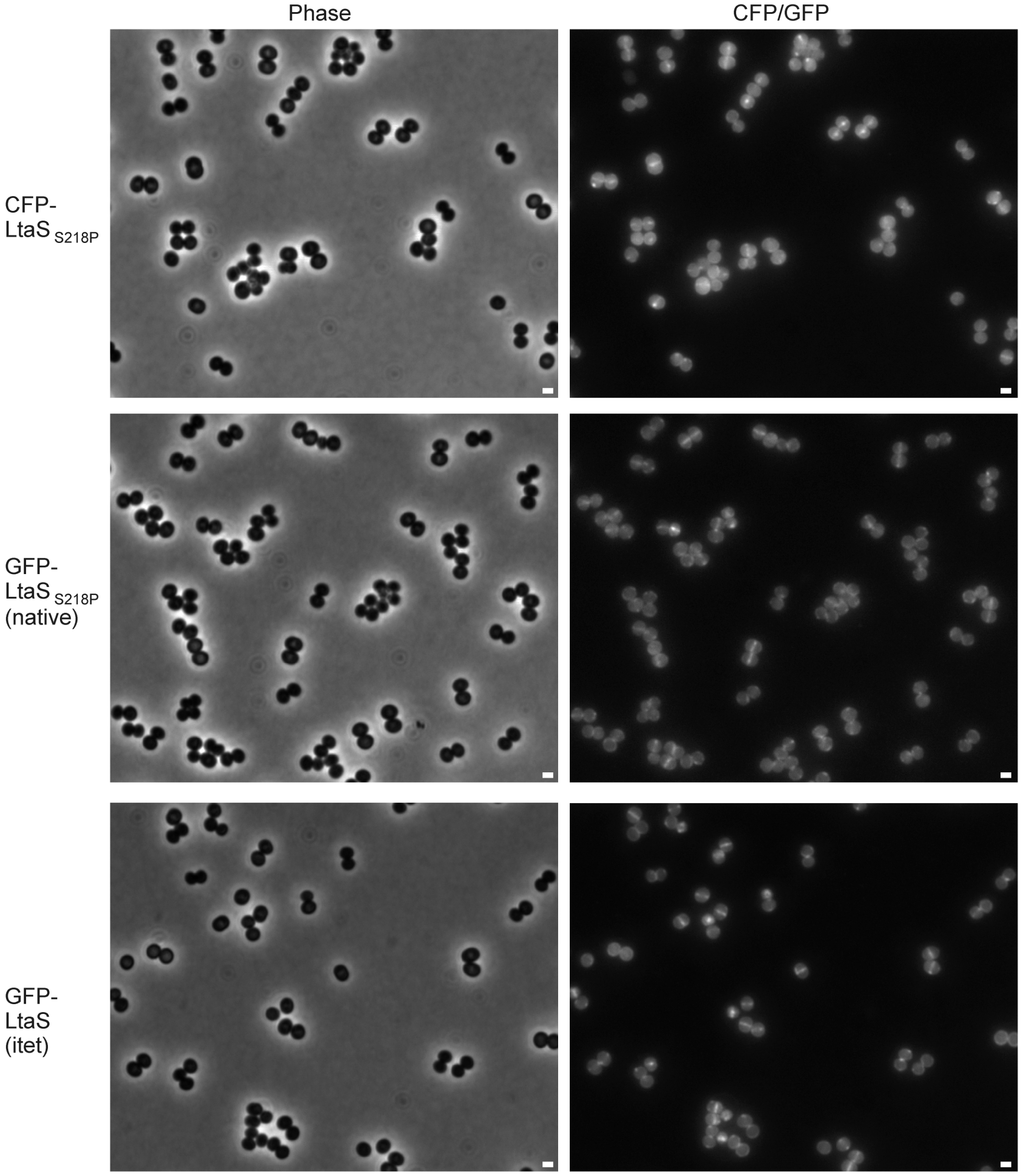

Fig. S5. Localization of LtaS in *S. aureus*. *S. aureus* LAC* strains containing pCL55-p*ltaS-cfp-ltaS*$_{S218P}$ (CFP-LtaS$_{S218P}$), pCL55-p*ltaS-gfp*$_{P7}$-*ltaS*$_{S218P}$ (GFP-LtaS$_{S218P}$ (native)) or p*itet-gfp*$_{P7}$-*ltaS*$_{S218P}$ (GFP-LtaS$_{S218P}$ (itet) grown in the presence of 200 ng/ml Atet) were grown to mid-exponential phase, mounted on a 1.2% PBS agarose slide and subsequently observed by fluorescence microscopy. Phase contrast (left) and fluorescence (right) images are shown. Scale bar = 1 μm.

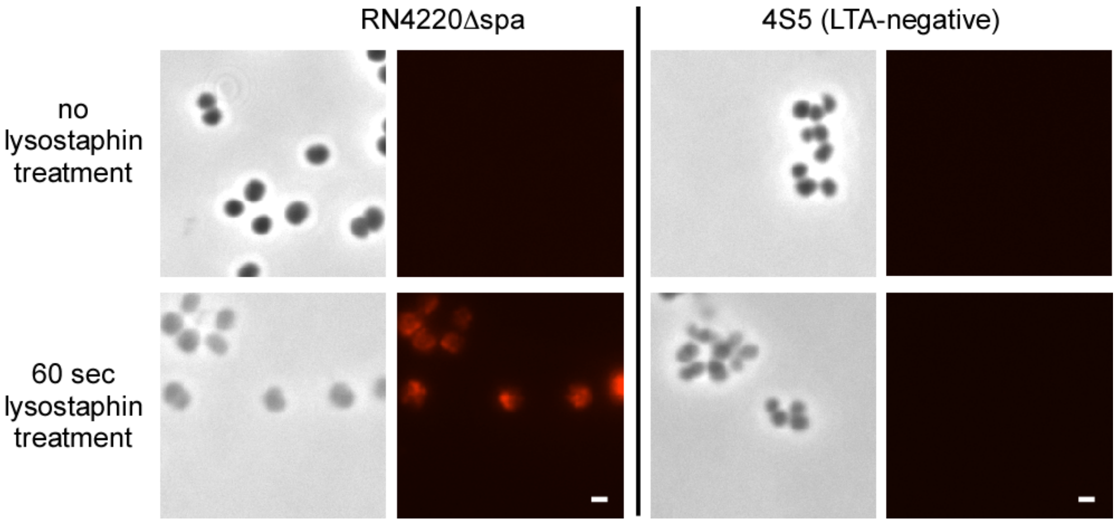

Fig. S6. Localization of LTA as assessed by immunofluorescence microscopy. *S. aureus* strain RN4220Δ*spa* and the isogenic LTA negative strain 4S5 were grown to mid-exponential phase and fixed as described in the material and method section. Where indicated cells were digested with a final concentration of 10 µg/ml lysostaphin for 60 sec. LTA was detected by incubating the cells with a mouse monoclonal polyglycerolphosphate specific primary antibody followed by incubation with anti-mouse Alexafluor 546 conjugated secondary antibody. Samples were subsequently observed by fluorescence microscopy. Scale bar = 1 µm.

Boles B.R., Thoendel M., Roth A.J., and Horswill A.R. (2010) Identification of genes involved in polysaccharide-independent *Staphylococcus aureus* biofilm formation. *PLoS One* **5:** e10146.

Charpentier E., Anton A.I., Barry P., Alfonso B., Fang Y., and Novick R.P. (2004) Novel cassette-based shuttle vector system for gram-positive bacteria. *App Environ Microbiol* **70:** 6076-6085.

Corrigan R.M., Abbott J.C., Burhenne H., Kaever V., and Gründling A. (2011) c-di-AMP is a new second messenger in *Staphylococcus aureus* with a role in controlling cell size and envelope stress. *PLoS Path* **7:** e1002217.

Duthie E.S., and Lorenz L.L. (1952) Staphylococcal coagulase; mode of action and antigenicity. *J Gen Microbiol* **6:** 95-107.

Fisher A.C., and DeLisa M.P. (2008) Laboratory evolution of fast-folding green fluorescent protein using secretory pathway quality control. *PLoS One* **3:** e2351.

Gründling A., and Schneewind O. (2007a) Genes required for glycolipid synthesis and lipoteichoic acid anchoring in *Staphylococcus aureus*. *J Bacteriol* **189:** 2521-2530.

Gründling A., and Schneewind O. (2007b) Synthesis of glycerol phosphate lipoteichoic acid in *Staphylococcus aureus*. *Proc Natl Acad Sci U S A* **104:** 8478-8483.

Karimova G., Dautin N., and Ladant D. (2005) Interaction network among *Escherichia coli* membrane proteins involved in cell division as revealed by bacterial two-hybrid analysis. *J Bacteriol* **187:** 2233-2243.

Karimova G., Pidoux J., Ullmann A., and Ladant D. (1998) A bacterial two-hybrid system based on a reconstituted signal transduction pathway. *Proc Natl Acad Sci U S A* **95:** 5752-5756.

Karimova G., Ullmann A., and Ladant D. (2001) Protein-protein interaction between *Bacillus stearothermophilus* tyrosyl-tRNA synthetase subdomains revealed by a bacterial two-hybrid system. *J Mol Microbiol Biotech* **3:** 73-82.

Lee C.Y., Buranen S.L., and Ye Z.H. (1991) Construction of single-copy integration vectors for *Staphylococcus aureus*. *Gene* **103:** 101-105.

Pereira P.M., Veiga H., Jorge A.M., and Pinho M.G. (2010) Fluorescent reporters for studies of cellular localization of proteins in *Staphylococcus aureus*. *App Environ Microbiol* **76:** 4346-4353.

Reed P., Veiga H., Jorge A.M., Terrak M., and Pinho M.G. (2011) Monofunctional transglycosylases are not essential for *Staphylococcus aureus* cell wall synthesis. *J Bacteriol* **193:** 2549-2556.

Sastalla I., Chim K., Cheung G.Y., Pomerantsev A.P., and Leppla S.H. (2009) Codon-optimized fluorescent proteins designed for expression in low-GC gram-positive bacteria. *App Environ Microbiol* **75:** 2099-2110.

Steele V.R., Bottomley A.L., Garcia-Lara J., Kasturiarachchi J., and Foster S.J. (2011) Multiple essential roles for EzrA in cell division of *Staphylococcus aureus*. *Mol Microbiol* **80:** 542-555.

Wörmann M.E., Reichmann N.T., Malone C.L., Horswill A.R., and Gründling A. (2011b) Proteolytic cleavage inactivates the *Staphylococcus aureus* lipoteichoic acid synthase. *J Bacteriol* **193:** 5279-5291.
